# Safety and Efficacy of Different Anticoagulant Doses for Patients with COVID-19 in the ICU: A Systematic Review and Meta-Analysis

**DOI:** 10.3390/jcm12062222

**Published:** 2023-03-13

**Authors:** Svetlana Rachina, Yuliya Belkova, Anastasia Shchendrygina, Aleksandr Suvorov, Denis Bourgeois, Marina Karuk, Violetta Sitnikova, Nikita Dyatlov

**Affiliations:** 1 Hospital Therapy Department No. 2, I.M. Sechenov First Moscow State Medical University, 119435 Moscow, Russia; 2 Department of Clinical Pharmacology, Smolensk State Medical University, 214019 Smolensk, Russia; 3 Institute of Biodesign and Complex Systems Modeling, I.M. Sechenov First Moscow State Medical University, 119435 Moscow, Russia; 4 Health Systemic Process (P2S), Research Unit UR4129, University Claude Bernard Lyon 1, University of Lyon, 69008 Lyon, France; denis.bourgeois@univ-lyon1.fr; 5 Endovascular Surgery Department, Russian Research Center of Surgery Named after Academician B.V. Petrovsky, 119991 Moscow, Russia

**Keywords:** COVID-19, ICU, thromboprophylaxis, anticoagulant, heparin, deep vein thrombosis, venous thromboembolism, pulmonary embolism

## Abstract

Critically ill COVID-19 patients have a high incidence of thromboembolic events, which significantly influence the risk of mortality. Anticoagulant therapy is generally recommended to these patients but the optimal dosing regimens require further investigations. The objective of this systematic review and meta-analysis was to assess the efficacy and safety of prophylactic, intermediate and therapeutic dose anticoagulation in COVID-19 patients admitted to the ICU. A systematic search for original prospective observational studies and clinical trials was performed in online databases from 2020 to 2022. A total of 13 studies (3239 patients) were included. The type of anticoagulant dosing showed no significant influence on short-term mortality (*p* = 0.84), deep vein thrombosis (*p* = 0.66), arterial thrombosis (*p* = 0.44), major bleeding (*p* = 0.35) and minor bleeding incidence (*p* = 0.46). An anticoagulation regimen significantly influenced pulmonary embolism occurrence (16% for prophylactic dose vs. 4% for therapeutic dose, *p* = 0.02), but the number of studies in the analysis was relatively low. In conclusion, the results of this meta-analysis suggest that critically ill COVID-19 patients admitted in the ICU have no benefit from therapeutic doses of anticoagulants and that all three thromboprophylaxis regimes have a comparable effect on short term mortality and venous thromboembolism incidence but for pulmonary embolism, for which the results were inconclusive.

## 1. Introduction

The novel coronavirus disease 2019 (COVID-19), caused by severe acute respiratory syndrome coronavirus 2 (SARS-CoV-2), is a major health problem worldwide [1]. The infection is associated with a range of phenotypes from asymptomatic or mild to severe disease and may result in respiratory or multiple organ failure and death [2,3].

Although SARS-CoV-2 virus primarily targets the respiratory system, in a large number of patients it causes cardiovascular injuries and hypercoagulation [4,5] commonly manifesting with venous thromboembolism (VTE) including pulmonary embolism (PE) and deep vein thrombosis (DVT) [6,7]. The highest risk of thrombotic and thromboembolic events has been observed for critically ill patients admitted to the intensive care units (ICU) [8,9]. The reported incidence of VTE or PE in the patients with severe COVID-19 is about 24–28% [10,11,12] and 22–33% [13,14,15], respectively. Meanwhile, patients with thrombotic complications in the ICU have increased risk of in-hospital mortality [16].

A body of evidence shows that anticoagulant therapy, especially with low molecular weight heparin, might improve the prognosis in COVID-19 patients [17,18,19]. In accordance with these findings, a number of international societies and regulators strongly recommend the administration of thromboprophylaxis in all hospitalized patients with COVID-19 [20,21,22]. At the same time, a non-negligible risk of venous thromboses remains in critically ill individuals even despite thromboprophylaxis [23,24], and the optimal dosing regimens of anticoagulants in this subgroup of patients are still controversial. As prophylactic doses of anticoagulants may not be effective for the prevention of thromboembolic events [25], intermediate to therapeutic doses are increasingly prescribed to COVID-19 patients admitted to the ICU despite a higher risk of bleeding complications [18,26] and possible lack of influence on in-hospital mortality [27].

Several meta-analyses have been performed to clarify the matter of optimal approach to thromboprophylaxis in critically ill COVID-19 patients [12,23,28,29], but still no definitive conclusion is available probably due to shortage of reliable evidence. Thus, many studies included in the analyses were retrospective. Given that the new prospective trials addressing the issue of thromboprophylaxis in ICU COVID-19 subjects, including randomized controlled trials (RCTs), have become available, it seems reasonable to reassess the whole range of data collected prospectively regarding the thromboprophylaxis in this subset of COVID-19 patients.

The objective of this systematic review and meta-analysis was to assess the efficacy and safety of prophylactic, intermediate and therapeutic dose anticoagulation in COVID-19 patients admitted to the ICU.

## 2. Materials and Methods

### 2.1. Data Sources and Search Strategy

This systematic review and meta-analysis were designed in accordance with the Preferred Reporting Items for Systematic Review and Meta-Analyses (PRISMA) guidelines (Supplementary Table S1) [30]. The registration number is INPLASY202320033.

A systematic search for eligible trials was performed in online databases including PubMed, EMBASE, Web of Science, Cochrane Library Database and Clinicaltrials.gov, databases of international and national respiratory and infectious diseases congresses by three independent investigators (Y.B., M.K. and V.S.) from 1 January 2020 to 1 June 2022. The search terms included the combination of the following key words: coronavirus disease 2019, SARS-CoV-2, COVID-19, intensive care unit, ICU, anticoagulation, anticoagulant, thromboprophylaxis, heparin, mortality, venous thromboembolism, deep vein thrombosis, deep venous thrombosis, pulmonary embolism, bleeding. No language filter was applied. Search strategy for the databases is presented in Supplementary Table S2.

### 2.2. Eligibility Criteria

Any original prospective observational study or clinical trial in any format was eligible for inclusion in the review irrespective of blinding and language.

The intended outcomes of this review and meta-analysis were: (1) short-term mortality (at the end of the follow-up period but no later than 30 days); (2) deep-vein thrombosis (DVT) incidence; (3) PE incidence; (4) arterial thrombosis (AT) incidence; (5) major bleeding incidence and (6) minor bleeding incidence. All outcomes were assessed separately. The publications that had reported other outcomes or the intended outcomes as a composite one were considered ineligible for inclusion (referenced as “without outcomes of interest” in the PRISMA diagram).

The inclusion criteria were: (1) enrolment of adults (age ≥ 18 years) with severe COVID-19 disease (SARS-CoV-2 infection confirmed by positive DNA test) who were hospitalized in the ICU and received at least one anticoagulation regimen (prophylactic dose, intermediate dose, therapeutic dose of low molecular weighted or unfractionated heparin). The definition of anticoagulation regimens is listed in Supplementary Table S3. The final two inclusion criteria included: (2) reporting on the outcomes of interests and (3) design of prospective observational study or clinical trial.

The exclusion criteria were as follows: (1) other types of publications (reviews, guidelines, commentaries, case reports, expert opinions, etc.); (2) identical data used in multiple reports (e.g., duplicated studies); (3) missing or insufficient data; (4) different study population, hospital site, intervention and/or outcomes.

### 2.3. Data Extraction and Data Synthesis

Three researchers (Y.B., M.K. and V.S.) independently searched and evaluated the titles, abstracts, and full texts for relevant studies and extracted data from eligible ones. All duplicated search results were excluded. Any disagreement was resolved through discussion between the researchers or by the decision of a fourth researcher (S.R.). Overall risk of confounding, selection and reporting bias was assessed with ROBINS Tool [31].

Predefined variables were extracted independently from each study as follows: (1) study information (study design, first author, title, journal, publication data and country); (2) characteristics of patients (age, gender, hospital unit, number of study participants); (3) interventions (administration of anticoagulants, type and dose of anticoagulants); (4) outcomes of interest (mortality, rates and types of thrombotic or bleeding events separately).

### 2.4. Statistical Analyses

Analysis was carried out in R v.4.1 using meta package [32]. Because a number of studies had only one arm, the frequency of the end point was chosen as a measure of the effect size. Individual trials were considered as random effects, heterogeneity was assessed using the inverse variance method and a restricted maximum-likelihood estimator (REML) was used to estimate $\tau^{2}$. The random-effects model was adopted for all the endpoints. The overall proportion was calculated with logit transformation and the Clopper–Pearson method was used to estimate confidence intervals. Dosage type was used for subgrouping for all the endpoints, for mortality the type of trial was also considered as a moderator and for venous thrombosis endpoint the usage of routine ultrasound was considered as a moderator. The effect size was estimated in each subgroup. Differences in effect size were assessed by Q test, assuming common $\tau^{2}$ in subgroups since the amount of trials is small [33]. Funnel plots were made for the overall groups and Egger’s test was performed to assess publication bias, where applicable. Sensitivity analysis was performed by the leave-one-out method.

## 3. Results

**Figure 1:**
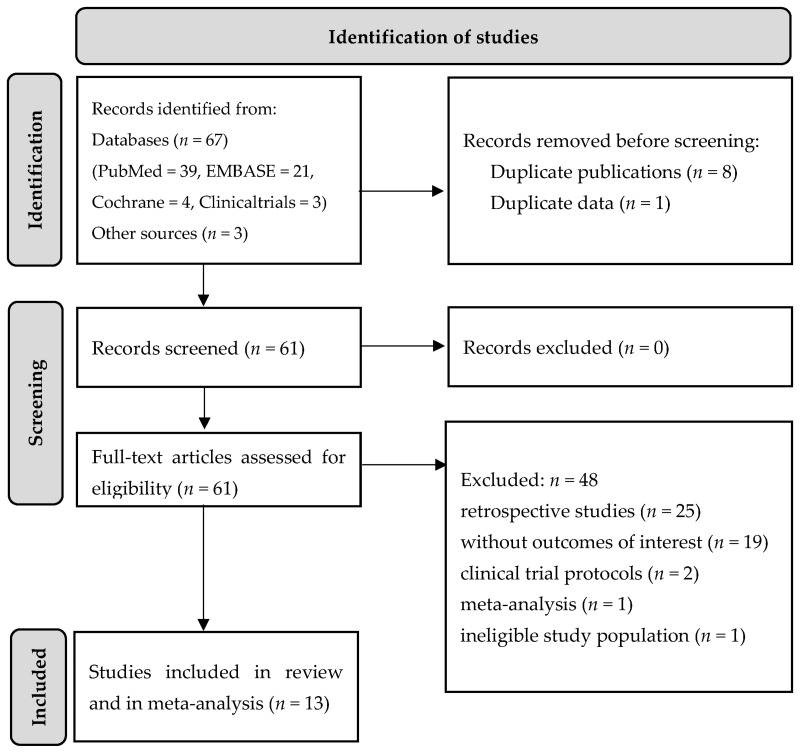
Preferred reporting items for systematic review and meta-analyses (PRISMA) flow diagram of literature screening.

A comprehensive search yielded 70 records, 13 of which met the inclusion and exclusion criteria for this systematic review (Figure 1). Of the 13 studies included in the meta-analysis, 5 were RCTs [34,35,36,37,38], 3 were before-after studies [39,40,41] and the remaining 5 were observational studies [10,42,43,44,45] (Table 1).

The results of the literature search and study selection are presented on the PRISMA flowchart (Figure 1) and in Supplementary Table S4.

To estimate the role of different anticoagulation dosing on the outcome “short-term mortality” 10 studies were available, for “DVT” 11 studies, for “PE” 4 studies, for “AT” 5 studies, for “major bleeding” 8 studies and for “minor bleeding” 5 studies were available. The number of studies and patients included in the assessment for each dosing regimen are presented in Table 2.

### 3.1. Short-Term Mortality

Ten studies (3106 patients) were included in the analysis of short-term mortality in COVID-19 patients admitted to the ICU. The assessment of prophylactic dose anticoagulation was performed in 10 studies, of intermediate dose in 3 studies and therapeutic dose in 4 studies. A summary of studies reporting short-term mortality among COVID-19 patients in ICU receiving anticoagulation is presented in Supplementary Table S5.

**Figure 2:**
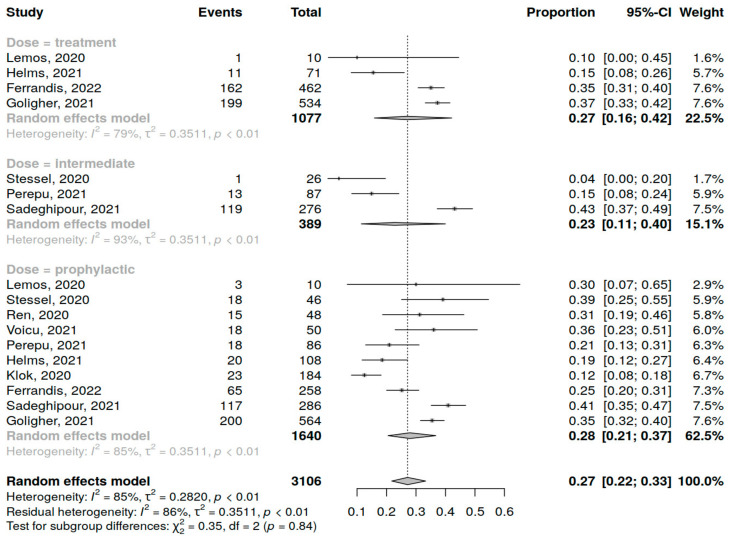
Forest plot of anticoagulation doses influence on short-term mortality in COVID-19 patients in the ICU [10,34,35,36,37,39,40,41,42,43].

The overall short-term mortality was 27% (95% CI 20; 34%) varying from study to study (range 12–42%) with high heterogeneity (I^2^ = 91%, *p* < 0.01) independent of the trial type (RCT vs. other) (*p* = 0.47) (Supplementary Figures S1 and S2). High heterogeneity was also observed inside each anticoagulant regimen group (Figure 2). The type of anticoagulant dosing showed no significant influence on short-term mortality (*p* = 0.84).

### 3.2. Deep Vein Thrombosis

Eleven studies (3134 patients) were included in the analysis of DVT incidence in COVID-19 patients in the ICU. The assessment of prophylactic dose anticoagulation was performed in 11 studies, of intermediate dose in 3 studies and therapeutic dose in 4 studies. A summary of studies reporting DVT incidence among COVID-19 patients in the ICU receiving anticoagulation is presented in Supplementary Table S6.

**Figure 3:**
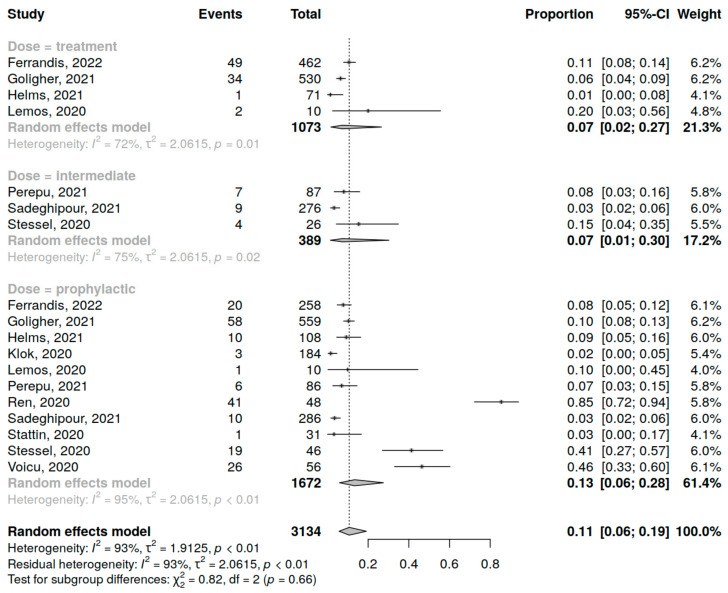
Forest plot of anticoagulation doses influence on DVT incidence in COVID-19 patients in the ICU [10,34,35,36,37,39,40,42,43,44,45].

The overall DVT incidence was 13% (95% CI 5; 28%), varying from study to study (range 2–85%) with high heterogeneity (I^2^ = 96%, *p* < 0.01). High heterogeneity was also observed inside each anticoagulant regimen group. The type of anticoagulant dosing showed no significant influence on DVT incidence in COVID-19 patients (*p* = 0.66) (Figure 3), whereas, in contrast, routine ultrasound examinations in all patients statistically significantly increased the rate of diagnosed DVT events (*p* < 0.01) (Supplementary Figures S3 and S4).

### 3.3. Pulmonary Embolism

Four studies (414 patients) were included in the analysis of PE incidence in COVID-19 patients in the ICU. The assessment of prophylactic dose anticoagulation was performed in 4 studies and of therapeutic dose in 2 studies. No included studies assessed the influence of an intermediate dose on PE occurrence. A summary of studies reporting PE among COVID-19 patients in ICU receiving anticoagulation is presented in Supplementary Table S7.

**Figure 4:**
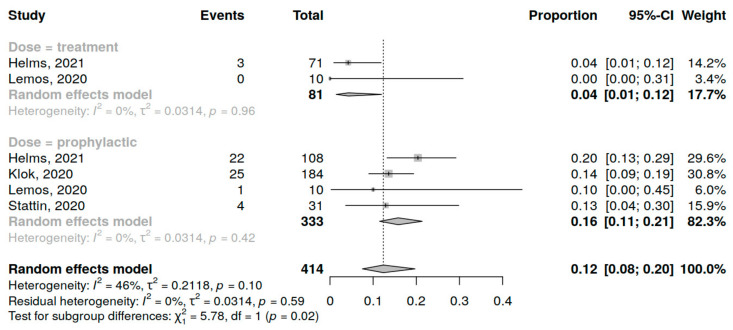
Forest plot of anticoagulation doses influence on PE incidence in COVID-19 patients in the ICU [10,35,39,44].

The overall PE incidence was 13% (95% CI 10; 17%), variations range from 5 to 14%, with low heterogeneity (I^2^ = 0%, *p* < 0.76) in total (Supplementary Figure S5) and for both dosing regimens. An anticoagulation regimen significantly influenced PE occurrence (16% for prophylactic dose vs. 4% for therapeutic dose, *p* = 0.02) (Figure 4), but the number of studies in the analysis was relatively low.

### 3.4. Arterial Thrombosis

Five studies (976 patients) were included in the analysis of AT incidence in COVID-19 patients in the ICU. The assessment of prophylactic dose anticoagulation was performed in 5 studies, of intermediate dose in 1 study and of therapeutic dose in 2 studies. A summary of studies reporting AT among COVID-19 patients in the ICU receiving anticoagulation is presented in Supplementary Table S8.

**Figure 5:**
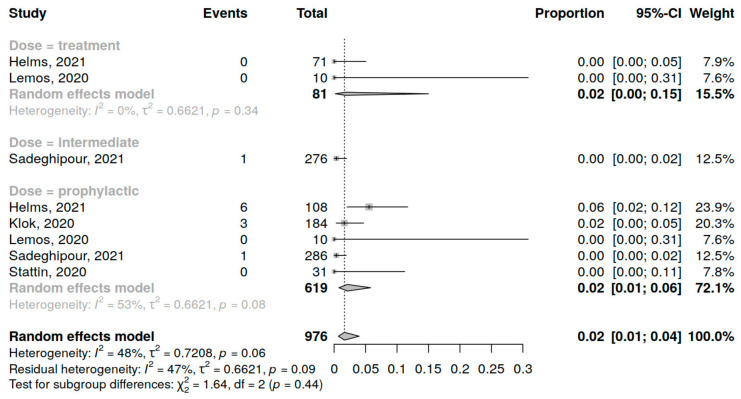
Forest plot of anticoagulation doses influence on AT incidence in COVID-19 patients in the ICU [10,35,37,39,44].

The overall arterial thrombosis incidence was 2% (95% CI 1; 4%), variations range from 0 to 3%, with moderate heterogeneity (I^2^ = 48%, *p* = 0.1) in total (Supplementary Figure S6) and for different dosing regimens. No significant influence of anticoagulant dosing regimen on AT incidence was shown (*p* = 0.44) (Figure 5).

### 3.5. Major Bleeding

Eight studies (2692 patients) were included in the analysis of major bleeding incidence in COVID-19 patients in the ICU. The assessment of prophylactic dose anticoagulation was performed in 7 studies, of intermediate dose in 2 studies and of therapeutic dose in 4 studies. A summary of studies reporting major bleeding among COVID-19 patients in the ICU receiving anticoagulation is presented in Supplementary Table S9.

**Figure 6:**
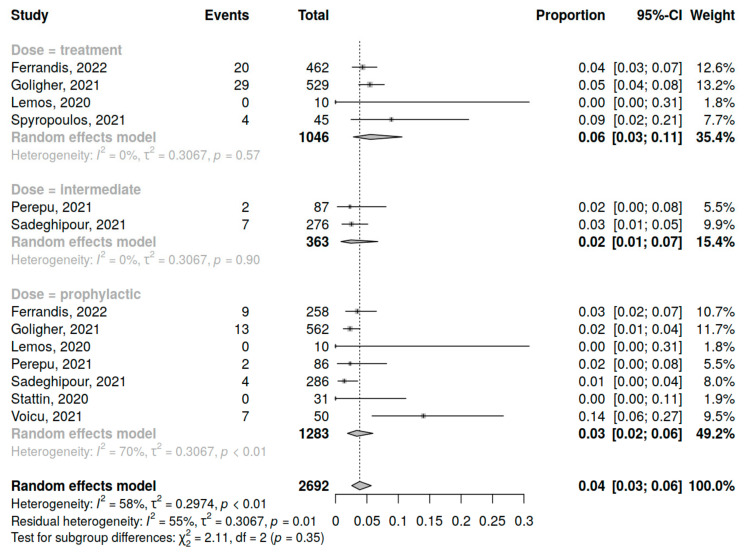
Forest plot of anticoagulation doses influence on major bleeding incidence in COVID-19 patients in the ICU [34,35,36,37,38,41,42,44].

The overall major bleeding incidence was 4% (95% CI 2; 7%) varying from study to study (range 0–14%) with high heterogeneity (I^2^ = 68%, *p* < 0.01) in total (Supplementary Figure S7) and for different dosing regimens. Although no significant influence of anticoagulant dosing regimen on major bleeding incidence was demonstrated (*p* = 0.35), there was a trend towards a higher rate of major bleeding occurrence in patients who had the treatment dose of anticoagulant (Figure 6).

### 3.6. Minor Bleeding

Five studies (1506 patients) were included in the analysis of minor bleeding incidence in COVID-19 patients in the ICU. The assessment of prophylactic dose anticoagulation was performed in 4 studies, of intermediate dose in 2 studies and of therapeutic dose in 3 studies. A summary of studies reporting minor bleeding among COVID-19 patients in the ICU receiving anticoagulation is presented in Supplementary Table S10.

**Figure 7:**
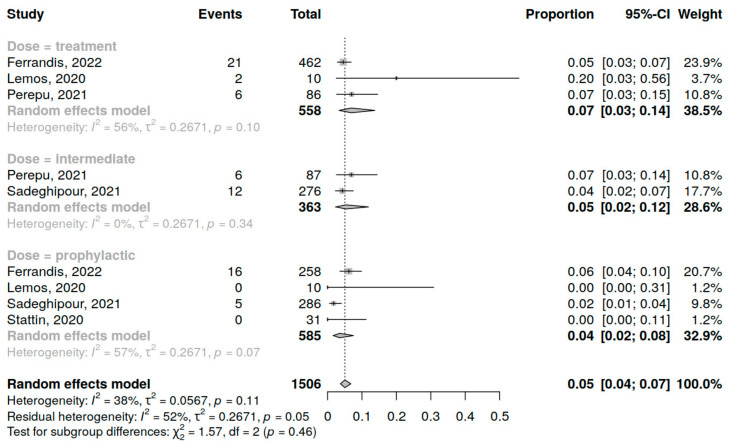
Forest plot of anticoagulation doses influence on minor bleeding incidence in COVID-19 patients in the ICU [35,36,37,42,44].

The overall major bleeding incidence was 5% (95% CI 3; 7%), varying from study to study (range 0–10%) with moderate heterogeneity (I^2^ = 46%, *p* = 0.11) in total (Supplementary Figure S8) and for different dosing regimens. No significant influence of anticoagulant dosing regimen on minor bleeding incidence was demonstrated (*p* = 0.46) (Figure 7).

### 3.7. Publication Bias and Sensitivity Analysis

ROBINS Tool was used to evaluate publication bias [31] and revealed a low-to-moderate risk of bias for both RCTs and observational studies (Supplementary Figure S9). Funnel plots for all outcomes were symmetric. Egger’s tests results evidenced a low probability of publication bias (Supplementary Figures S10–S15). A sensitivity analysis by the means of the leave-one-out method (Supplementary Figures S16–S21) showed similar results to those in the main study.

## 4. Discussion

In this systematic review and meta-analysis, the results from 13 high-quality studies including 3239 critically ill COVID-19 patients admitted in the ICU demonstrated comparable effect of different thromboprophylaxis regimes (prophylactic, intermediate or therapeutic) on short-term mortality and incidence of thromboembolic events, but for PE, with a similar safety profile. To our knowledge, this is the first meta-analysis which specifically focuses on a cohort of critically ill COVID-19 patients aiming to assess the efficacy and safety of different types of anticoagulant dosing in prospective studies only, including RCTs.

It is appreciated that thromboembolic events are just one of several determinates of death in these patients’ population. Nevertheless, it has been shown that the incidence of COVID-19-related thrombosis in the ICU is as high as 28% [49] and associated with significantly higher odds of mortality (OR 1.74, 95% CI, 1.01–2.98) [50]. Based on preliminary reports showing no benefit of high doses of thromboprophylaxis in critically ill COVID-19 subjects, the prophylactic dose is generally recommended [20,51]. However, the question regarding an efficient dose regimen in severe COVID-19 patients remains to be further investigated.

We found that the overall mortality rate in ICU COVID-19 individuals was 27%. Despite inclusion of high-quality studies, a heterogeneity of mortality was high and could not be explained either by the type of antithrombotic dosing or by the study design. One can assume that the heterogeneity might be a result of the difference in disease severity across the studies. Unfortunately, there is not any reliable marker to compare the severity of COVID-19 disease in the selected studies. If we consider the percentage of those that required mechanical ventilation, there is a risk of bias as we appreciate the access to this kind of treatment was limited during the pandemic and largely dependent on local settings rather than the patient’s conditions. The data on disease severity assessed by a scoring system such as APACHE [46] or SAPS scores [47,48] were reported in 6 and 3 studies, respectively. APACHE scores ranged from 8 to 16 across the studies, paradoxically demonstrating that those with the lowest APACHE score had a higher mortality rate at about 40% [37].

According to our sub-group analysis, short-term mortality did not differ significantly depending on the regimen of thromboprophylaxis: prophylactic, intermediate or therapeutic. Few meta-analyses have been highlighted on this topic when performing a sub-group analysis of critically ill individuals. Yasuda et al. [29], similarly, did not observe any significant difference in short-term mortality between groups of severe COVID-19 patients on prophylactic and treatment doses of anticoagulants. In other meta-analyses by Loffewdo et al. [52] and Elsebaie et al. [53] the finding was confirmed. Parisi et al. [54], however, reported a reduction in mortality rates in those on full-dose anticoagulation, but in this study in-hospital mortality was assessed. In addition, the conclusion was based on the analysis of 4 retrospective studies. Therefore, our results confirmed previously reported trends detected in sub-group analyses, that in critically ill COVID-19 patients therapeutic regimes cause no clear benefit over other types of anticoagulant dosing. One might find the data counterintuitive. However, the findings might confirm the assumption that thrombi formation in critically ill COVID-19 subjects has an immune mechanism due to endotelitis and, therefore, is resistant to antithrombotic treatment of any dose [55].

In our study the pooled incidence of VTE was 13% for both DVT and PE. AT occurred in 2%. Types of anticoagulant dosing did not influence the occurrence of VTE and AT. Of note, the incidence of PE was significantly higher in those on prophylactic doses compared to those on therapeutic doses. Although this finding might seem to be rational, it should be interpreted with caution as the number of studies reported PE outcomes were low (2 for therapeutic and 4 for prophylactic doses). Our findings are in line with the results of a recent meta-analysis by Valeriani et al. [12], in which the data of 4 RCTs that included critically ill COVID-19 patients were pooled. The study showed no reduction in incidence of VTE in those on full-dose anticoagulants compared to low dose. The beneficial effect of a high-dose regimen on VTE incidence in an ICU subgroup was not confirmed either in the study by Vedovati et al. [56].

Oppositely, when Yasuda et al. [29] pooled the data of 5 RCTs and observational studies they reported lower VTE incidence in those with severe COVID-19 on therapeutic doses of anticoagulant compared to a prophylactic one. However, the criteria for severity of COVID-19 were not thoroughly defined in this study, and therefore it was not clear if those individuals were critically ill patients. Previously, it has been shown that VTE was significantly reduced by full doses of anticoagulants in hospitalized non-critically ill subjects [12]. Therefore, interference could not be ruled out. The only meta-analysis reported data on AT incidence in cohorts of interest and did not demonstrate any influence of the thromboprophylaxis regime on AT occurrence [52].

The incidence of major and minor bleeding was comparable across all three types of thromboprophylaxis regimes. A trend towards the higher rate of major bleeding events in those on a therapeutic dose was observed. In those on a therapeutic dose, pooled incidence of major bleeding accounted for 6% (4 studies, 1046 patients, I^2^ = 0%), followed by 3% in subjects who received a prophylactic dose (7 studies, 363 patients, I^2^ = 70%) and 2% in patients on an intermediate dose (2 studies, 1676 patients, I^2^ = 95%); however, subgroup differences were not significant. Similarly, in the study by Valeriani and colleagues [12] the incidence of the risk of major bleeding did not differ between the groups of ICU COVID-19 patients on high and low doses of anticoagulants. Yasuda and colleagues [29] reported opposite findings, demonstrating the association of treatment dose with increased risk of major bleeding in a cohort of severe COVID-19 patients, but as it has been previously mentioned the criteria of disease severity were not defined clearly.

The overall results of our meta-analysis showed that all three doses of antithrombotic treatment administrated in critically ill COVID-19 individuals had a comparable effect on short-term mortality, VTE and AT incidence, as well as similar bleeding risk. We also observed a trend towards lower incidence of PE in those on therapeutic doses which, however, might occur at the cost of higher bleeding risk. Nevertheless, our study adds to the previous findings. This meta-analysis for the first time looked specifically at the cohort of critically ill COVID-19 patients in the prospective studies, while in previous publications [12,23,28,29,53] these patients were analyzed as a subgroup based on the set of prospective and retrospective data. We also incorporated recent studies not included in previous reviews [41,42]. We believe that our findings will be of value not only to improve thromboprophylaxis in the current ICU patients with SARS-CoV-2 infection but also in case of future pandemics caused by this or similar viruses.

We appreciate our study has some limitations such as variation in dosage and duration of the antithrombotic treatment across the studies, diversity in types of reported outcomes and lack of reliable criteria for disease severity assessment. Another possible limitation is the small sample size for some outcomes, particularly for intermediate doses because the occurrence outcomes had not been separately reported. For this review we have not contacted the corresponding authors of manuscripts with missing data which is also a limitation of the publication. We acknowledge that some data could have possibly been missed from the analysis. Ongoing and future studies can also bring new data and new perspectives to anticoagulant prophylaxis in critically ill COVID-19 patients. Therefore, our findings should be considered with caution.

In conclusion, the results of this meta-analysis suggest that critically ill COVID-19 patients admitted in the ICU have no benefit from therapeutic doses of anticoagulants and that all three thromboprophylaxis regimes have a comparable effect on short term mortality and VTE incidence but for PE, for which the results were inconclusive. Given the trends toward increased bleeding complications in those on full-dose anticoagulation, the results of our study do not support intermediate-to-therapeutic anticoagulant prophylaxis in the ICU COVID-19 patients. At the same time, future studies, some of which have already been published in 2023, can bring new perspectives to anticoagulant prophylaxis in critically ill COVID-19 patients.

## Figures and Tables

**Table 1:** Characteristics of studies included in meta-analysis.

Study (Author, Year)	Country	Study Design	Patients in ICU, %	Comparisons	Type of Anticoagulation	Sample, *n*	Mean/Median Age, y	Male, %	Average APACHE II Score [46]	Outcomes					
										Short-Term Mortality	DVT	PE	AT	Major Bleeding	Minor Bleeding
Ferrandis, 2022 [42]	Spain	Observational	100	Treatment dose	UFH	462	63.5	32	12	+	+			+	+
				Prophylactic dose	UFH	258	63	33	12						
Goligher, 2021 [34]	Canada, multinational	Open-label RCT	100	Treatment dose	UFH LMWH	534	60.4	72.2	14	+	+			+	
				Prophylactic dose	UFH LMWH	564	61.7	67.9	13						
Helms, 2021 [39]	France	Before/after study	100	Treatment dose	LMWH, UFH	71	64	66.2	47 *	+	+	+	+		
				Prophylactic dose	LMWH, UFH	108	61	76.9	48 *						
Klok, 2020 [10]	Norway	Observational	100	Prophylactic dose	LMWH (nadroparin)	184	64	76	na	+	+	+	+		
Lemos, 2020 [35]	Brazil	Open-label RCT	100	Treatment dose	LMWH (enoxaparin)	10	55	90	56 **	+	+	+	+	+	+
				Prophylactic dose	LMWH (enoxaparin)	10	58	70	56 **						
Perepu, 2021 [36]	USA	Multi-center, open-label RCT	62	Intermediate dose	LMWH (enoxaparin)	86	65	54	na	+	+			+	+
				Prophylactic dose	LMWH (enoxaparin)	87	63.5	58	na						
Ren, 2020 [43]	China	Cross-sectional	100	Prophylactic dose	LMWH	48	70	54.2	16	+	+				
Sadeghipour, 2021 [37]	Iran	Multicenter RCT with a 2 × 2 factorial design	100	Intermediate dose	LMWH (enoxaparin)	276	62	57	8	+	+		+	+	+
				Prophylactic dose	LMWH (enoxaparin)	286	61	58.7	8						
Spyropoulos, 2021 [38]	USA	RCT	100	Treatment dose	LMWH (enoxaparin)	45	65.8	52.7	na					+	
Stattin, 2020 [44]	Sweden	Observational	100	Prophylactic dose	LMWH (dalteparin)	31	65	81	53 **		+	+	+	+	+
Stessel, 2020 [40]	Belgium	Longitudinal controlled before/after study	100	Intermediate dose	LMWH (nadroparin)	26	62	57.7	11	+	+				
				Prophylactic dose	LMWH (nadroparin)	46	69.5	73.9	13						
Voicu, 2020 [45]	France	Observational	100	Prophylactic dose	LMWH (enoxaparin), UFH	56	na	75	na		+				
Voicu, 2021 [41]	France	Before/after observational exploratory study	100	Prophylactic dose	LMWH (enoxaparin), UFH	50	62	72	na	+				+	

RCT, randomized controlled trial; LMWH, low molecular weight heparin; UFH, unfractionated heparin; DVT, deep vein thrombosis; PE, pulmonary embolism; AT, arterial thrombosis; na, not available. * assessed by the SAPS II Score [47], ** assessed by the SAPS III Score [48].

**Table 2:** The number of studies and patients included in meta-analysis for different dosing regimens.

Outcome	Prophylactic Dose		Intermediate Dose		Therapeutic Dose		Total	
	No. of Studies	No. of Patients	No. of Studies	No. of Patients	No. of Studies	No. of Patients	No. of Studies	No. of Patients
Short-term mortality	10	1640	3	389	4	1077	10	3106
Deep vein thrombosis	11	1672	3	389	4	1073	11	3134
Pulmonary embolism	4	333	0	0	2	81	4	414
Arterial thrombosis	5	619	1	276	2	81	5	976
Major bleeding	7	1283	2	363	4	1046	8	2692
Minor bleeding	4	585	2	363	3	558	5	1506

## Data Availability

The datasets used and/or analyzed during the current study are available from the corresponding authors on reasonable request.

## Supplementary Materials

The following supporting information can be downloaded at: https://www.mdpi.com/article/10.3390/jcm12062222/s1.
