# Supplementary material for: Safety and Efficacy of Different Anticoagulant Doses for Patients with COVID-19 in the ICU: A Systematic Review and Meta-Analysis
Source: J Clin Med. 2023 Mar 13;12(6):2222. doi: 10.3390/jcm12062222 (PMC10057479; doi:10.3390/jcm12062222)
Supplement: Supplementary file 1 [file jcm-12-02222-s001.zip › jcm-2230519-supplementary.pdf]

SUPPLEMENTARY MATERIALS

Table of Contents

Supplementary Table S1. PRISMA checklist. 2

Supplementary Table S2. Search strategy for the databases....................5

Supplementary Table S3. Definition of dosing regimens of anticoagulants. 6

Supplementary Table S4. The main reasons for excluding articles from the systematic review and meta-analysis....................6

Supplementary Table S5. Summary of studies reporting short-term mortality among COVID-19 patients in ICU receiving anticoagulation....................11

Supplementary Figure S1. Forest plot of short-term mortality in COVID-19 patients in the ICU. 11

Supplementary Figure S2. Forest plot of trial type influence on short-term mortality in COVID-19 patients in the ICU....................12

Supplementary Table S6. Summary of studies reporting DVT among COVID-19 patients in ICU receiving anticoagulation. 12

Supplementary Figure S3. Forest plot of DVT incidence in COVID-19 patients in the ICU. 13

Supplementary Figure S4. Forest plot of routine ultrasound investigation influence on the frequency of diagnosed DVT events in COVID-19 patients in the ICU....................13

Supplementary Table S7. Summary of studies reporting PE among COVID-19 patients in ICU receiving anticoagulation. 13

Supplementary Figure S5. Forest plot of PE incidence in COVID-19 patients in the ICU. 14

Supplementary Table S8. Summary of studies reporting AT among COVID-19 patients in ICU receiving anticoagulation. 14

Supplementary Figure S6. Forest plot of AT incidence in COVID-19 patients in the ICU....................14

Supplementary Table S9. Summary of studies reporting major bleeding among COVID-19 patients in ICU receiving anticoagulation. 14

Supplementary Figure S7. Forest plot of major bleeding incidence in COVID-19 patients in the ICU. 15

Supplementary Table S10. Summary of studies reporting minor bleeding among COVID-19 patients in ICU receiving anticoagulation. 15

Supplementary Figure S8. Forest plot of minor bleeding incidence in COVID-19 patients in the ICU. 15

Supplementary Figure S9. Risk-of-bias plots for RCTs (A) and observational studies (B). 16

Supplementary Figure S10. Funnel plot and Egger's test results for the studies of short-term mortality in COVID-19 patients in the ICU. 17

Supplementary Figure S11. Funnel plot and Egger's test results for the studies of DVT incidence in COVID-19 patients in the ICU. 17

Supplementary Figure S12. Funnel plot and Egger's test results for the studies of PE incidence in COVID-19 patients in the ICU....................18

Supplementary Figure S13. Funnel plot and Egger's test results for the studies of AT incidence in COVID-19 patients in the ICU....................18

Supplementary Figure 14. Funnel plot and Egger's test results for the studies of major bleeding in COVID-19 patients in the ICU. 19

Supplementary Figure S15. Funnel plot and Egger's test results for the studies of minor bleeding in COVID-19 patients in the ICU. 19
Supplementary Figure S16. Sensitivity analysis of anticoagulation doses influence on short-term mortality in COVID-19 patients in the ICU by the means of the leave-one-out method. 20
Supplementary Figure S17. Sensitivity analysis of anticoagulation doses influence on DVT incidence in COVID-19 patients in the ICU by the means of the leave-one-out method. 20
Supplementary Figure S18. Sensitivity analysis of anticoagulation doses influence on PE incidence in COVID-19 patients in the ICU by the means of the leave-one-out method. 20
Supplementary Figure S19. Sensitivity analysis of anticoagulation doses influence on AT incidence in COVID-19 patients in the ICU by the means of the leave-one-out method. 20
Supplementary Figure S20. Sensitivity analysis of anticoagulation doses influence on major bleeding incidence in COVID-19 patients in the ICU by the means of the leave-one-out method. 21
Supplementary Figure S21. Sensitivity analysis of anticoagulation doses influence on minor bleeding incidence in COVID-19 patients in the ICU by the means of the leave-one-out method. 21

Supplementary Table S1. PRISMA checklist.

**Section and Topic**	**Item #**	**Checklist item**	**Location where item is reported, pp.**
**TITLE**			
Title	1	Identify the report as a systematic review.	1
**ABSTRACT**			
Abstract	2	See the PRISMA 2020 for Abstracts checklist.	1
**INTRODUCTION**			
Rationale	3	Describe the rationale for the review in the context of existing knowledge.	1-2
Objectives	4	Provide an explicit statement of the objective(s) or question(s) the review addresses.	2
**METHODS**			
Eligibility criteria	5	Specify the inclusion and exclusion criteria for the review and how studies were grouped for the syntheses.	2-3
Information sources	6	Specify all databases, registers, websites, organisations, reference lists and other sources searched or consulted to identify studies. Specify the date when each source was last searched or consulted.	2
Search strategy	7	Present the full search strategies for all databases, registers and websites, including any filters and limits used.	Suppl. 5-6
Selection process	8	Specify the methods used to decide whether a study met the inclusion criteria of the review, including how many reviewers screened each record and each report retrieved, whether they worked independently, and if applicable, details of automation	3

Section and Topic	Item #	Checklist item	Location where item is reported, pp.
		tools used in the process.	
Data collection process	9	Specify the methods used to collect data from reports, including how many reviewers collected data from each report, whether they worked independently, any processes for obtaining or confirming data from study investigators, and if applicable, details of automation tools used in the process.	3
Data items	10a	List and define all outcomes for which data were sought. Specify whether all results that were compatible with each outcome domain in each study were sought (e.g. for all measures, time points, analyses), and if not, the methods used to decide which results to collect.	3
	10b	List and define all other variables for which data were sought (e.g. participant and intervention characteristics, funding sources). Describe any assumptions made about any missing or unclear information.	3
Study risk of bias assessment	11	Specify the methods used to assess risk of bias in the included studies, including details of the tool(s) used, how many reviewers assessed each study and whether they worked independently, and if applicable, details of automation tools used in the process.	3
Effect measures	12	Specify for each outcome the effect measure(s) (e.g. risk ratio, mean difference) used in the synthesis or presentation of results.	3
Synthesis methods	13a	Describe the processes used to decide which studies were eligible for each synthesis (e.g. tabulating the study intervention characteristics and comparing against the planned groups for each synthesis (item #5)).	3
	13b	Describe any methods required to prepare the data for presentation or synthesis, such as handling of missing summary statistics, or data conversions.	3
	13c	Describe any methods used to tabulate or visually display results of individual studies and syntheses.	3
	13d	Describe any methods used to synthesize results and provide a rationale for the choice(s). If meta-analysis was performed, describe the model(s), method(s) to identify the presence and extent of statistical heterogeneity, and software package(s) used.	3
	13e	Describe any methods used to explore possible causes of heterogeneity among study results (e.g. subgroup analysis, meta-regression).	3
	13f	Describe any sensitivity analyses conducted to assess robustness of the synthesized results.	3
Reporting bias assessment	14	Describe any methods used to assess risk of bias due to missing results in a synthesis (arising from reporting biases).	3

Section and Topic	Item #	Checklist item	Location where item is reported, pp.
Certainty assessment	15	Describe any methods used to assess certainty (or confidence) in the body of evidence for an outcome.	3
**RESULTS**			
Study selection	16a	Describe the results of the search and selection process, from the number of records identified in the search to the number of studies included in the review, ideally using a flow diagram.	3-4
	16b	Cite studies that might appear to meet the inclusion criteria, but which were excluded, and explain why they were excluded.	Suppl. 5-10
Study characteristics	17	Cite each included study and present its characteristics.	15-16
Risk of bias in studies	18	Present assessments of risk of bias for each included study.	Suppl. 15
Results of individual studies	19	For all outcomes, present, for each study: (a) summary statistics for each group (where appropriate) and (b) an effect estimate and its precision (e.g. confidence/credible interval), ideally using structured tables or plots.	Suppl. 10-15
Results of syntheses	20a	For each synthesis, briefly summarise the characteristics and risk of bias among contributing studies.	5-9
	20b	Present results of all statistical syntheses conducted. If meta-analysis was done, present for each the summary estimate and its precision (e.g. confidence/credible interval) and measures of statistical heterogeneity. If comparing groups, describe the direction of the effect.	5-9
	20c	Present results of all investigations of possible causes of heterogeneity among study results.	5-9
	20d	Present results of all sensitivity analyses conducted to assess the robustness of the synthesized results.	9, Suppl. 19-20
Reporting biases	21	Present assessments of risk of bias due to missing results (arising from reporting biases) for each synthesis assessed.	Suppl. 15-18
Certainty of evidence	22	Present assessments of certainty (or confidence) in the body of evidence for each outcome assessed.	5-9
**DISCUSSION**			
Discussion	23a	Provide a general interpretation of the results in the context of other evidence.	9-11
	23b	Discuss any limitations of the evidence included in the review.	11
	23c	Discuss any limitations of the review processes used.	11
	23d	Discuss implications of the results for practice, policy, and future research.	11
**OTHER INFORMATION**			

Section and Topic	Item #	Checklist item	Location where item is reported, pp.
Registration and protocol	24a	Provide registration information for the review, including register name and registration number, or state that the review was not registered.	2
	24b	Indicate where the review protocol can be accessed, or state that a protocol was not prepared.	2
	24c	Describe and explain any amendments to information provided at registration or in the protocol.	2
Support	25	Describe sources of financial or non-financial support for the review, and the role of the funders or sponsors in the review.	11
Competing interests	26	Declare any competing interests of review authors.	11
Availability of data, code and other materials	27	Report which of the following are publicly available and where they can be found: template data collection forms; data extracted from included studies; data used for all analyses; analytic code; any other materials used in the review.	11

Supplementary Table S2. Search strategy for the databases.

**PubMed**		
1	heparin[Title/Abstract] OR enoxaparin[Title/Abstract] OR clexane[Title/Abstract] OR fondaparinux[Title/Abstract] OR LMWH[Title/Abstract] OR nadroparin[Title/Abstract] OR dalteparin[Title/Abstract]	88,811
2	thrombo-prophylaxis[Title/Abstract] OR thromboprophylaxis[Title/Abstract]	6,198
3	anti-thrombotic[Title/Abstract] OR antithrombotic[Title/Abstract] OR anticoagulant*[Title/Abstract] OR anticoagulat*[Title/Abstract] OR anti-coagulant*[Title/Abstract] OR anticoagulat*[Title/Abstract] OR antithrombotic[Title/Abstract]	131,285
4	VTE[Title/Abstract] OR "venous thromboembolism"[Title/Abstract] OR mortality[Title/Abstract] OR "deep vein thrombosis"[Title/Abstract] OR DVT[Title/Abstract] OR "arterial thrombosis" [Title/Abstract] OR "pulmonary embolism"[Title/Abstract] OR bleeding[Title/Abstract]	1,213,468
5	covid-19[Title/Abstract] OR SARS-cov2[Title/Abstract] OR "novel coronavirus"[Title/Abstract] OR "COVID 19"[Title/Abstract]	298,448
6	"intensive care"[Title/Abstract] OR ICU[Title/Abstract] OR "critically ill"[Title/Abstract]	243,043
7	prospective*[Title/Abstract] OR trial[Title/Abstract] OR randomized[Title/Abstract] OR cohort[Title/Abstract] OR observational[Title/Abstract]	2,425,879
8	#1 OR #2 OR #3	198,205
9	#4 AND #5 AND #6 AND#7 AND#8	**39**
**EMBASE**		
	(heparin OR enoxaparin OR clexane OR fondaparinux OR LMWH OR nadroparin OR dalteparin OR LMWH OR thromboprophylaxis OR anti-thrombotic OR antithrombotic OR	**21**

	anticoagulant OR) AND (VTE OR "venous thromboembolism" OR mortality OR "deep vein thrombosis" OR DVT OR "pulmonary embolism" OR "arterial thrombosis" OR bleeding) AND (COVID-19 OR SARS-cov2 OR "novel coronavirus" OR "COVID 19") AND ("intensive care" OR ICU OR "critically ill") AND "clinical trial"	
**Cochrane library**		
1	heparin OR enoxaparin OR clexane OR fondaparinux OR LMWH OR nadroparin OR dalteparin OR LMWH	17,611
2	thrombo-prophylaxis OR thromboprophylaxis	1,058
3	anti-thrombotic OR antithrombotic OR anticoagulant* OR anticoagulat* OR anti-coagulant* OR anticoagulat* OR antithrombotic	14,349
4	#1 OR #2 OR #3	23,222
5	VTE OR "venous thromboembolism" OR mortality OR "deep vein thrombosis" OR DVT OR "pulmonary embolism" OR "arterial thrombosis" OR bleeding	17,211
6	COVID-19 OR SARS-cov2 OR "novel coronavirus" OR "COVID 19"	958
7	"intensive care" OR ICU OR "critically ill"	56,558
6	#4 AND #5 AND #6 AND#7 (Limit to trials)	**4**
**Clinicaltrials.gov**		
	Condition: COVID-19 OR SARS-COV OR coronavirus<br>Other terms: anticoagulant OR heparin OR enoxaparin OR clexane OR fondaparinux OR LMWH OR nadroparin OR dalteparin OR LMWH OR antithrombotic OR thromboprophylaxis<br>Status: Completed	**3**
	**Total**	**67**

Supplementary Table S3. Definition of dosing regimens of anticoagulants.

Anticoagulant	Prophylactic dose	Intermediate dose	Therapeutic dose
Enoxaparin	40 mg QD<br>30 mg QD	1 mg/kg QD<br>0.5 mg/kg BID	100 IU (1 mg)/kg BID but no more than 100 mg
Dalteparin	2,000-10,000 QD	-	-
Fondaparinux	2.5 mg QD		
Nadroparin	2,850 – 5,700 IU QD	3,600 - IU BID	-
Unfractionated heparin	5,000 IU TID<br>200 IU/kg QD (creatinine clearance <30 mL/min)	-	10,000-15,000 IU QD<br>500 IU/kg QD (creatinine clearance <30 mL/min)

Supplementary Table S4. The main reasons for excluding articles from the systematic review and meta-analysis.

N	Study reference	Reason for exclusion
1.	Aljuhani O., Al Sulaiman K., Hafiz A., Eljaaly K., Alharbi A., Algarni R., Al Homaid S., Kahtani K., Alsulaiman T., Vishwakarma R., et al. Comparison between standard Vs. Escalated dose venous thromboembolism (DVT) prophylaxis in critically ill patients with COVID-19: A two centers, observational study. Saudi Pharm J. 2022 Apr;30(4):398-406. doi: 10.1016/j.jsps.2022.01.022.	Ineligible study design. Retrospective cohort study.

2.	Al-Samkari H., Gupta S., Leaf R.K., Wang W., Rosovsky R.P., Brenner S.K., Hayek S.S., Berlin H., Kapoor R., Shaefi S., et al. Thrombosis, Bleeding, and the Observational Effect of Early Therapeutic Anticoagulation on Survival in Critically Ill Patients With COVID-19. Ann Intern Med. 2021 May;174(5):622-632. doi: 10.7326/M20-6739.	No data about outcomes of interest.
3.	Artifoni M., Danic G., Gautier G., Gicquel P., Boutoille D., Raffi F., Néel A., Lecomte R. Systematic assessment of venous thromboembolism in COVID-19 patients receiving thromboprophylaxis: incidence and role of D-dimer as predictive factors. J Thromb Thrombolysis. 2020 Jul;50(1):211-216. doi: 10.1007/s11239-020-02146-z.	Ineligible study design. Retrospective cohort study.
4.	Atallah B., Sadik Z.G., Salem N., El Nekidy W.S., Almahmeed W., Park W.M., Cherfan A., Hamed F., Mallat J. The impact of protocol-based high-intensity pharmacological thromboprophylaxis on thrombotic events in critically ill COVID-19 patients. Anaesthesia. 2021 Mar;76(3):327-335. doi: 10.1111/anae.15300.	Ineligible study design. Retrospective cohort study.
5.	Avruscio G., Camporese G., Campello E., Bernardi E., Persona P., Passarella C., Noventa F., Cola M., Navalesi P., Cattelan A., et al. COVID-19 and Venous Thromboembolism in Intensive Care or Medical Ward. Clin Transl Sci. 2020 Nov;13(6):1108-1114. doi: 10.1111/cts.12907.	No data about outcomes of interest.
6.	Beun R., Kusadasi N., Sikma M., Westerink J., Huisman A. Thromboembolic events and apparent heparin resistance in patients infected with SARS-CoV-2. Int J Lab Hematol. 2020 Jun;42 Suppl 1(Suppl 1):19-20. doi: 10.1111/ijlh.13230.	Ineligible study design. Description of clinical cases.
7.	Bikdeli B., Talasaz A.H., Rashidi F., Bakhshandeh H., Rafiee F., Rezaeifar P., Baghizadeh E., Matin S., Jamalkhani S., Tahamtan O., et al. Intermediate-Dose versus Standard-Dose Prophylactic Anticoagulation in Patients with COVID-19 Admitted to the Intensive Care Unit: 90-Day Results from the INSPIRATION Randomized Trial. Thromb Haemost. 2022 Jan;122(1):131-141. doi: 10.1055/a-1485-2372.	Duble publication.
8.	Busani S., Tosi M., Mighali P., Vandelli P., D'Amico R., Marietta M., Forfori F., Donati A., Cinnella G., De Monte A., et al. Multi-centre, three arm, randomized controlled trial on the use of methylprednisolone and unfractionated heparin in critically ill ventilated patients with pneumonia from SARS-CoV-2 infection: A structured summary of a study protocol for a randomised controlled trial. Trials. 2020 Aug 17;21(1):724. doi: 10.1186/s13063-020-04645-z.	Ineligible study design. Clinical trial protocol.
9.	Canoglu K., Saylan B. Therapeutic dosing of low-molecular-weight heparin may decrease mortality in patients with severe COVID-19 infection. Ann Saudi Med. 2020 Nov-Dec;40(6):462-468. doi: 10.5144/0256-4947.2020.462.	Ineligible study design. Retrospective cohort study.
10.	Chen S., Zhang D., Zheng T., Yu Y., Jiang J. DVT incidence and risk factors in critically ill patients with COVID-19. J Thromb Thrombolysis. 2021 Jan;51(1):33-39. doi: 10.1007/s11239-020-02181-w.	Ineligible study design. Retrospective cohort study.
11.	Desborough M.J.R., Doyle A.J., Griffiths A., Retter A., Breen K.A., Hunt B.J. Image-proven thromboembolism in patients with severe COVID-19 in a tertiary critical care unit in the United Kingdom. Thromb Res. 2020 Sep;193:1-4. doi: 10.1016/j.thromres.2020.05.049.	No data about outcomes of interest.
12.	Dujardin R.W.G., Hilderink B.N., Haksteen W.E., Middeldorp S., Vlaar A.P.J., Thachil J., Müller M.C.A., Juffermans N.P. Biomarkers for the prediction of venous thromboembolism in critically ill COVID-19 patients. Thromb Res. 2020 Dec;196:308-312. doi: 10.1016/j.thromres.2020.09.017.	No data about outcomes of interest.

13.	Elsebaie M.A.T., Baral B., Elsebaie M., Shrivastava T., Weir C., Kumi D., Birch N.W. Does High-Dose Thromboprophylaxis Improve Outcomes in COVID-19 Patients? A Meta-analysis of Comparative Studies. TH Open. 2022 Oct 19;6(4):e323-e334. doi: 10.1055/a-1930-6492.	Ineligible study design. Meta-analysis.
14.	Ferguson J., Volk S., Vondracek T., Flanigan J., Chernaik A. Empiric Therapeutic Anticoagulation and Mortality in Critically Ill Patients With Respiratory Failure From SARS-CoV-2: A Retrospective Cohort Study. J Clin Pharmacol. 2020 Nov;60(11):1411-1415. doi: 10.1002/jcph.1749.	Ineligible study design. Retrospective cohort study.
15.	Fernández-Capitán C., Barba R., Díaz-Pedroche M.D.C., Sigüenza P., Demelo-Rodriguez P., Siniscalchi C., Pedrajas J.M., Farfán-Sedano A.I., Olivera P.E., Gómez-Cuervo C., et al. Presenting Characteristics, Treatment Patterns, and Outcomes among Patients with Venous Thromboembolism during Hospitalization for COVID-19. Semin Thromb Hemost. 2021 Jun;47(4):351-361. doi: 10.1055/s-0040-1718402.	Ineligible study design. Retrospective cohort study.
16.	Fogarty H., Townsend L., Ni Cheallaigh C., Bergin C., Martin-Loeches I., Browne P., Bacon C.L., Gaule R., Gillett A., Byrne M., et al. COVID19 coagulopathy in Caucasian patients. Br J Haematol. 2020 Jun;189(6):1044-1049. doi: 10.1111/bjh.16749.	No data about outcomes of interest.
17.	Ierardi A.M., Coppola A., Fusco S., Stellato E., Aliberti S., Andrisani M.C., Vespro V., Arrichiello A., Panigada M., Monzani V., et al. Early detection of deep vein thrombosis in patients with coronavirus disease 2019: who to screen and who not to with Doppler ultrasound? J Ultrasound. 2021 Jun;24(2):165-173. doi: 10.1007/s40477-020-00515-1.	No data about outcomes of interest.
18.	Jonmarker S., Hollenberg J., Dahlberg M., Stackelberg O., Litorell J., Everhov Å.H., Järnbert-Pettersson H., Söderberg M., Grip J., Schandl A., et al. Dosing of thromboprophylaxis and mortality in critically ill COVID-19 patients. Crit Care. 2020 Nov 23;24(1):653. doi: 10.1186/s13054-020-03375-7.	Ineligible study design. Retrospective cohort study.
19.	Jonmarker S., Litorell J., Dahlberg M., Stackelberg O., Everhov Å.H., Söderberg M., Rubenson-Wahlin R., Günther M., Mårtensson J., Hollenberg J., et al. An observational study of intermediate- or high-dose thromboprophylaxis for critically ill COVID-19 patients. Acta Anaesthesiol Scand. 2022 Mar;66(3):365-374. doi: 10.1111/aas.14013.	Ineligible study design. Retrospective cohort study.
20.	Kharma N., Roehrig S., Shible A.A., Elshafei M.S., Osman D., Elsaid I.M., Mustafa S.F., Aldabi A., Smain O.A.M., Lance M.D. Anticoagulation in critically ill patients on mechanical ventilation suffering from COVID-19 disease, The ANTI-CO trial: A structured summary of a study protocol for a randomised controlled trial. Trials. 2020 Sep 7;21(1):769. doi: 10.1186/s13063-020-04689-1.	No data about outcomes of interest.
21.	Lavinio A., Ercole A., Battaglini D., Magnoni S., Badenes R., Taccone F.S., Helbok R., Thomas W., Pelosi P., Robba C., et al. Safety profile of enhanced thromboprophylaxis strategies for critically ill COVID-19 patients during the first wave of the pandemic: observational report from 28 European intensive care units. Crit Care. 2021 Apr 22;25(1):155. doi: 10.1186/s13054-021-03543-3.	Ineligible study design. Retrospective cohort study.
22.	Levi M., Thachil J., Iba T., Levy J.H. Coagulation abnormalities and thrombosis in patients with COVID-19. Lancet Haematol. 2020 Jun;7(6):e438-e440. doi: 10.1016/S2352-3026(20)30145-9.	Ineligible study design. Retrospective cohort study.
23.	Lins P.R.G., de Albuquerque C.C.C., Assis C.F., Rodrigues B.C.D., E Siqueira Campos B.P., de Oliveira Valle E., Cabrera C.P.S., de Oliveira Gois J., Segura G.C., Strufaldi F.L., et al. Cov-hep study: heparin in standard anticoagulation based on citrate for continuous veno-venous	Ineligible study design. Clinical trial protocol.

	hemodialysis in patients with COVID-19: a structured summary of a study protocol for a randomized controlled trial. Trials. 2020 Nov 11;21(1):920. doi: 10.1186/s13063-020-04814-0.	
24.	Llitjos J.F., Leclerc M., Chochois C., Monsallier J.M., Ramakers M., Auvray M., Merouani K. High incidence of venous thromboembolic events in anticoagulated severe COVID-19 patients. J Thromb Haemost. 2020 Jul;18(7):1743-1746. doi: 10.1111/jth.14869.	Ineligible study design. Retrospective cohort study.
25.	Lodigiani C., Iapichino G., Carenzo L., Cecconi M., Ferrazzi P., Sebastian T., Kucher N., Studt J.D., Sacco C., Bertuzzi A., et al. Venous and arterial thromboembolic complications in COVID-19 patients admitted to an academic hospital in Milan, Italy. Thromb Res. 2020 Jul;191:9-14. doi: 10.1016/j.thromres.2020.04.024.	Ineligible study design. Retrospective cohort study.
26.	Maatman T.K., Jalali F., Feizpour C., Douglas A. 2nd, McGuire S.P., Kinnaman G., Hartwell J.L., Maatman B.T., Kreutz R.P., Kapoor R., et al. Routine Venous Thromboembolism Prophylaxis May Be Inadequate in the Hypercoagulable State of Severe Coronavirus Disease 2019. Crit Care Med. 2020 Sep;48(9):e783-e790. doi: 10.1097/CCM.0000000000004466.	Ineligible study design. Retrospective cohort study.
27.	Martinelli I., Ciavarella A., Abbattista M., Aliberti S., De Zan V., Folli C., Panigada M., Gori A., Artoni A., Ierardi A.M., et al. Increasing dosages of low-molecular-weight heparin in hospitalized patients with Covid-19. Intern Emerg Med. 2021 Aug;16(5):1223-1229. doi: 10.1007/s11739-020-02585-9.	Ineligible study population.
28.	Mennuni M.G., Renda G., Grisafi L., Rognoni A., Colombo C., Lio V., Foglietta M., Petrilli I., Pirisi M., Spinoni E., et al. Clinical outcome with different doses of low-molecular-weight heparin in patients hospitalized for COVID-19. J Thromb Thrombolysis. 2021 Oct;52(3):782-790. doi: 10.1007/s11239-021-02401-x.	Ineligible study design. Retrospective cohort study.
29.	Middeldorp S., Coppens M., van Haaps T.F., Foppen M., Vlaar A.P., Müller M.C.A, Bouman C.C.S, Beenen L.F.M., Kootte R.S., Heijmans J., et al. Incidence of venous thromboembolism in hospitalized patients with COVID-19. J Thromb Haemost. 2020 Aug;18(8):1995-2002. doi: 10.1111/jth.14888.	Ineligible study design. Retrospective cohort study.
30.	Nahum J., Morichau-Beauchant T., Daviaud F., Echegut P., Fichet J., Maillet J.M., Thierry S. Venous Thrombosis Among Critically Ill Patients With Coronavirus Disease 2019 (COVID-19). JAMA Netw Open. 2020 May 1;3(5):e2010478. doi: 10.1001/jamanetworkopen.2020.10478.	Ineligible study design. Retrospective cohort study.
31.	NCT04367831	No data about outcomes of interest.
32.	NCT04390074	No data about outcomes of interest.
33.	NCT04730856	No data about outcomes of interest.
34.	Panigada M., Bottino N., Tagliabue P., Grasselli G., Novembrino C., Chantarangkul V., Pesenti A., Peyvandi F., Tripodi A. Hypercoagulability of COVID-19 patients in intensive care unit: A report of thromboelastography findings and other parameters of hemostasis. J Thromb Haemost. 2020 Jul;18(7):1738-1742. doi: 10.1111/jth.14850.	No data about outcomes of interest.
35.	Paranjpe I., Fuster V., Lala A., Russak A.J., Glicksberg B.S., Levin M.A., Charney A.W., Narula J., Fayad Z.A., Bagiella E., et al. Association of Treatment Dose Anticoagulation With In-Hospital Survival Among	Ineligible study design. Letter to

	Hospitalized Patients With COVID-19. J Am Coll Cardiol. 2020 Jul 7;76(1):122-124. doi: 10.1016/j.jacc.2020.05.001.	editor. No data about outcomes of interest.
36.	Pavoni V., Gianesello L., Pazzi M., Stera C., Meconi T., Frigieri F.C. Venous thromboembolism and bleeding in critically ill COVID-19 patients treated with higher than standard low molecular weight heparin doses and aspirin: A call to action. Thromb Res. 2020 Dec;196:313-317. doi: 10.1016/j.thromres.2020.09.013.	Ineligible study design. Retrospective cohort study.
37.	Poissy J., Goutay J., Caplan M., Parmentier E., Duburcq T., Lassalle F., Jeanpierre E., Rauch A., Labreuche J., Susen S., et al. Pulmonary Embolism in Patients With COVID-19: Awareness of an Increased Prevalence. Circulation. 2020 Jul 14;142(2):184-186. doi: 10.1161/CIRCULATIONAHA.120.047430.	Ineligible study design. Letter to editor. No data about outcomes of interest.
38.	Salisbury R., Iotchkova V., Jaafar S., Morton J., Sangha G., Shah A., Untiveros P., Curry N., Shapiro S. Incidence of symptomatic, image-confirmed venous thromboembolism following hospitalization for COVID-19 with 90-day follow-up. Blood Adv. 2020 Dec 22;4(24):6230-6239. doi: 10.1182/bloodadvances.2020003349.	Ineligible study design. Retrospective cohort study.
39.	Søvik S., Bådstøløkken P.M., Sørensen V., Myhre P.L., Prebensen C., Omland T., Berdal J.E. A single-centre, prospective cohort study of COVID-19 patients admitted to ICU for mechanical ventilatory support. Acta Anaesthesiol Scand. 2021 Mar;65(3):351-359. doi: 10.1111/aas.13726.	No data about outcomes of interest.
40.	Spiezia L., Boscolo A., Poletto F., Cerruti L., Tiberio I., Campello E., Navalesi P., Simioni P. COVID-19-Related Severe Hypercoagulability in Patients Admitted to Intensive Care Unit for Acute Respiratory Failure. Thromb Haemost. 2020 Jun;120(6):998-1000. doi: 10.1055/s-0040-1710018.	Ineligible study design. Retrospective cohort study.
41.	Tang N., Bai H., Chen X., Gong J., Li D., Sun Z. Anticoagulant treatment is associated with decreased mortality in severe coronavirus disease 2019 patients with coagulopathy. J Thromb Haemost. 2020 May;18(5):1094-1099. doi: 10.1111/jth.14817.	Ineligible study design. Retrospective cohort study.
42.	Tang X., Du R.H., Wang R., Cao T.Z., Guan L.L., Yang C.Q., Zhu Q., Hu M., Li X.Y., Li Y., et al. Comparison of Hospitalized Patients With ARDS Caused by COVID-19 and H1N1. Chest. 2020 Jul;158(1):195-205. doi: 10.1016/j.chest.2020.03.032.	No data about outcomes of interest.
43.	Thomas W., Varley J., Johnston A., Symington E., Robinson M., Sheares K., Lavinio A., Besser M. Thrombotic complications of patients admitted to intensive care with COVID-19 at a teaching hospital in the United Kingdom. Thromb Res. 2020 Jul;191:76-77. doi: 10.1016/j.thromres.2020.04.028.	Ineligible study design. Retrospective cohort study.
44.	Torres-Machorro A., Anguiano-Álvarez V.M., Grimaldo-Gómez F.A., Rodríguez-Zanella H., Cortina de la Rosa E., Mora-Canela S., Lerma C., García-Cruz E., Ramos-Enriquez Á., Ramirez-Marroquin S.,et al. Asymptomatic deep vein thrombosis in critically ill COVID-19 patients despite therapeutic levels of anti-Xa activity. Thromb Res. 2020 Dec;196:268-271. doi: 10.1016/j.thromres.2020.08.043.	No data about outcomes of interest.
45.	Trigonis R.A., Holt D.B., Yuan R., Siddiqui A.A., Craft M.K., Khan B.A., Kapoor R., Rahman O. Incidence of Venous Thromboembolism in Critically Ill Coronavirus Disease 2019 Patients Receiving Prophylactic Anticoagulation. Crit Care Med. 2020 Sep;48(9):e805-e808. doi: 10.1097/CCM.0000000000004472.	Ineligible study design. Retrospective cohort study.
46.	Trinh M., Chang D., Govindarajulu U., Kane E., Fuster V., Kohli-Seth R., Ahmed S., Levin M.A., Martin D. Therapeutic Anticoagulation Is	No data about outcomes of interest.

	Associated with Decreased Mortality in Mechanically Ventilated COVID-19 Patients. medRxiv. doi: 10.1101/2020.05.30.20117929. 2020.05.30.20117929	
47.	Ugur M., Adiyeke E., Recep E., Bakan N., Yiyit N. Aggressive Thromboprophylaxis Improves Clinical Process and Decreases the Need of Intensive Care Unit in Covid-19. Pak J Med Sci. 2021 May-Jun;37(3):668-674. doi: 10.12669/pjms.37.3.3687.	Ineligible study design. Retrospective cohort study.
48.	Volteas P., Drakos P., Alkadaa L.N., Cleri N.A., Asencio A.A., Oganov A., Giannopoulos S., Saadon J.R., Mikell C.B. 3rd, Rubano J.A., et al. Low-molecular-weight heparin compared with unfractionated heparin in critically ill COVID-19 patients. J Vasc Surg Venous Lymphat Disord. 2022 Sep;10(5):1128-1136. doi: 10.1016/j.jvsv.2022.04.019.	Ineligible study design. Retrospective cohort study.
49.	Wang Y., Lu X., Li Y., Chen H., Chen T., Su N., Huang F., Zhou J., Zhang B., Yan F., et al. Clinical Course and Outcomes of 344 Intensive Care Patients with COVID-19. Am J Respir Crit Care Med. 2020 Jun 1;201(11):1430-1434. doi: 10.1164/rccm.202003-0736LE.	No data about outcomes of interest.

Supplementary Table S5. Summary of studies reporting short-term mortality among COVID-19 patients in ICU receiving anticoagulation.

Study (author, year)	RCT	Proportion of patients with 30-day mortality (n/N)		
		Prophylactic dose	Intermediate dose	Therapeutic dose
Ferrandis, 2022	no	65/258		162/462
Goligher, 2021	yes	200/564		199/534
Helms, 2021	no	20/108		11/71
Klok, 2020	no	23/184		
Lemos, 2020	yes	3/10		1/10
Perepu, 2021	yes	18/86	13/87	
Ren, 2020	no	15/48		
Sadeghipour, 2021	yes	117/286	119/276	
Stessel, 2020	no	18/46	1/26	
Voicu, 2021	no	18/50		

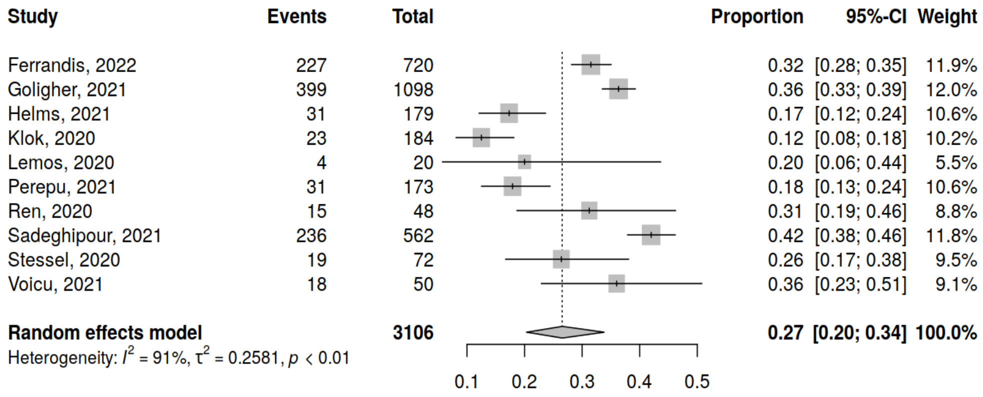

Supplementary Figure S1. Forest plot of short-term mortality in COVID-19 patients in the ICU.

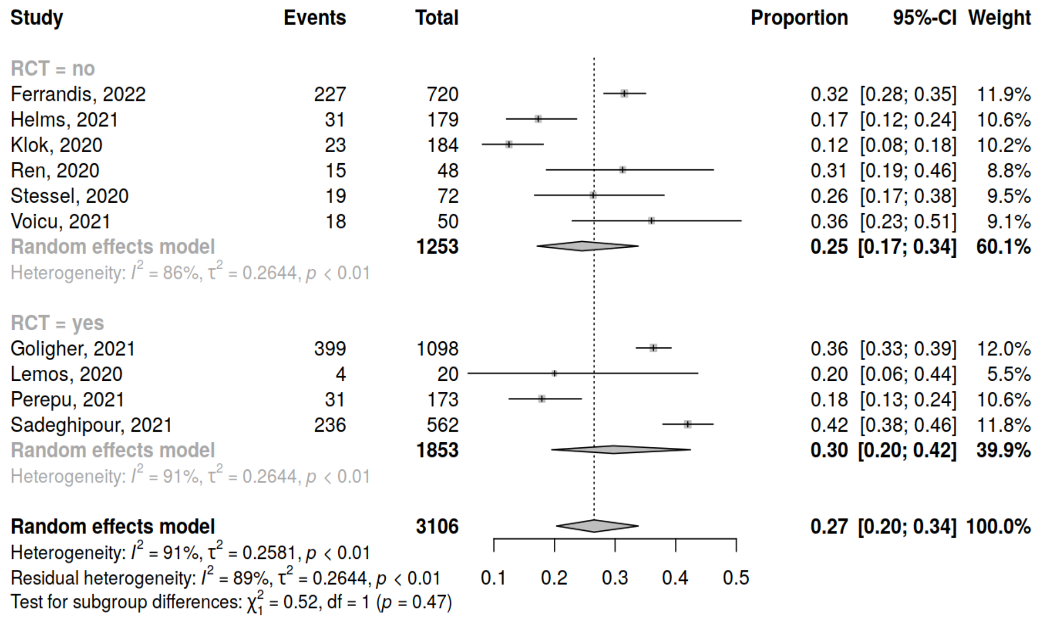

Supplementary Figure S2. Forest plot of trial type influence on short-term mortality in COVID-19 patients in the ICU.

Supplementary Table S6. Summary of studies reporting DVT among COVID-19 patients in ICU receiving anticoagulation.

Study (author, year)	Routine ultrasound to diagnose DVT	Proportion of patients with DVT (n/N)		
		Prophylactic dose	Intermediate dose	Therapeutic dose
Ferrandis, 2022	na	20/258		49/462
Goligher, 2021	no	58/559		34/530
Helms, 2021	no	10/108		1/71
Klok, 2020	no	3/184		
Lemos, 2020	no	1/10		2/10
Perepu, 2021	na	6/86	7/87	
Ren, 2020	yes	41/48		
Sadeghipour, 2021	no	10/286	9/276	
Stattin, 2020	yes	1/31		
Stessel, 2020	yes	19/46	4/26	
Voicu, 2020	yes	26/56		

na – not available

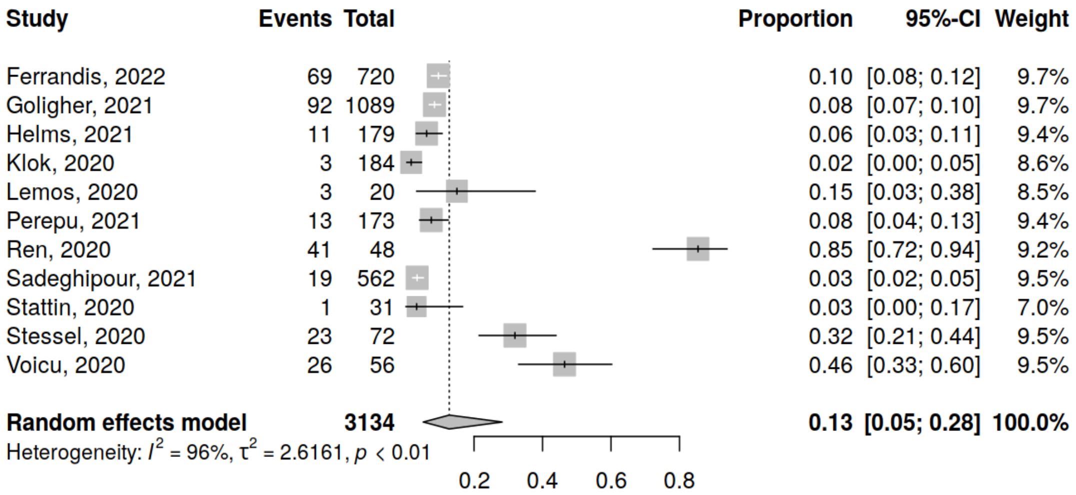

Supplementary Figure S3. Forest plot of DVT incidence in COVID-19 patients in the ICU.

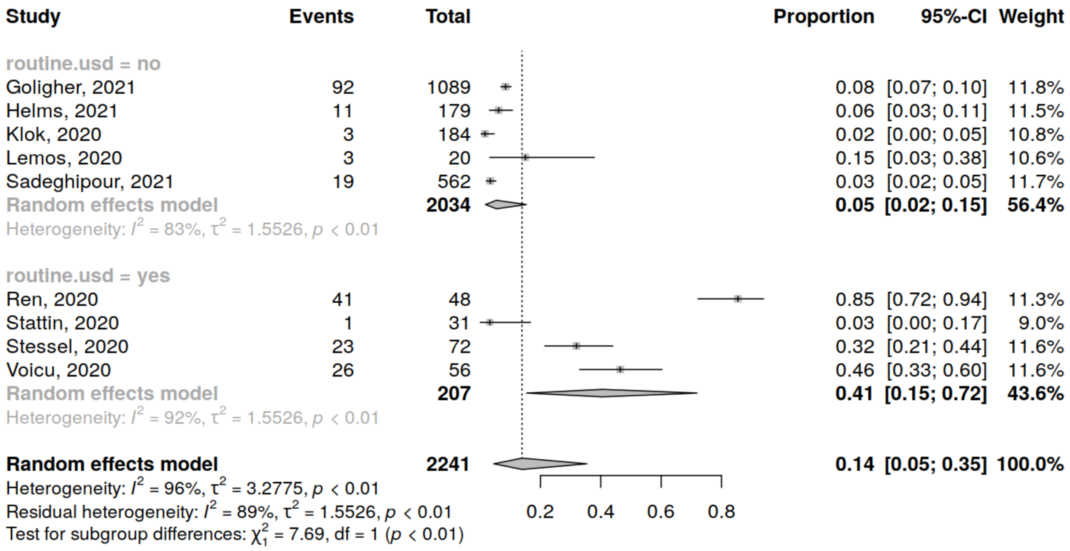

Supplementary Figure S4. Forest plot of routine ultrasound investigation influence on the frequency of diagnosed DVT events in COVID-19 patients in the ICU.

Supplementary Table S7. Summary of studies reporting PE among COVID-19 patients in ICU receiving anticoagulation.

Study (author, year)	Proportion of patients with PE (n/N)		
	Prophylactic dose	Intermediate dose	Therapeutic dose
Helms, 2021	22/108		3/71
Klok, 2020	25/184		
Lemos, 2020	1/10		0/10
Stattin, 2020	4/31		

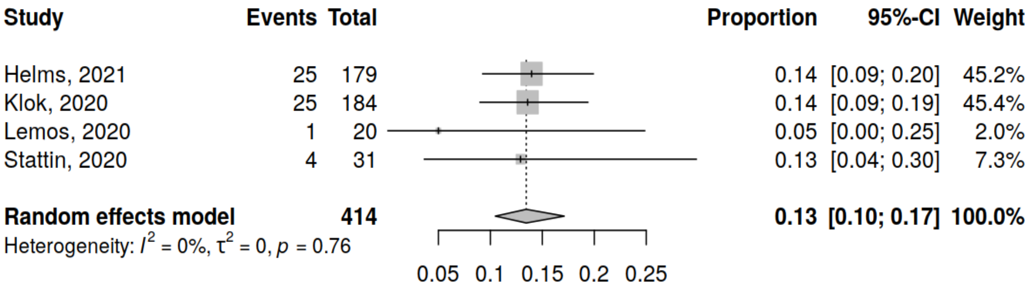

Supplementary Figure S5. Forest plot of PE incidence in COVID-19 patients in the ICU.

Supplementary Table S8. Summary of studies reporting AT among COVID-19 patients in ICU receiving anticoagulation.

Study<br>Author, year	Proportion of patients with AT (n/N)		
	Prophylactic dose	Intermediate dose	Therapeutic dose
Helms, 2021	6/108		0/71
Klok, 2020	3/184		
Lemos, 2020	0/10		0/10
Sadeghipour, 2021	1/286	1/276	
Stattin, 2020	0/31		

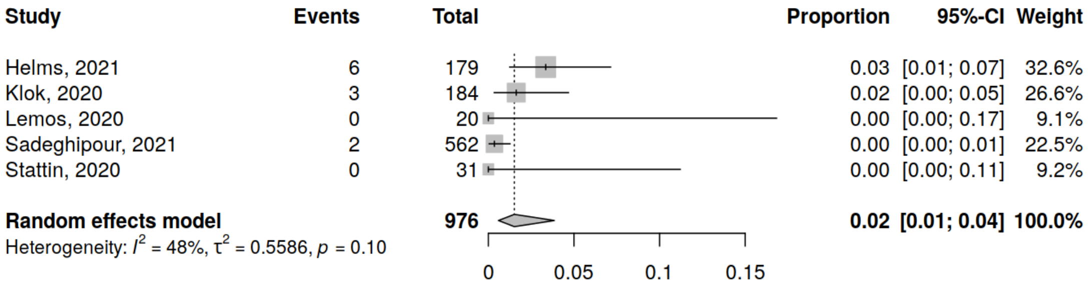

Supplementary Figure S6. Forest plot of AT incidence in COVID-19 patients in the ICU.

Supplementary Table S9. Summary of studies reporting major bleeding among COVID-19 patients in ICU receiving anticoagulation.

Study (author, year)	Proportion of patients with major bleeding (n/N)		
	Prophylactic dose	Intermediate dose	Therapeutic dose
Ferrandis, 2022	9/258		20/462
Goligher, 2021	13/562		29/529
Lemos, 2020	0/10		0/10
Perepu, 2021	2/86	2/87	
Sadeghipour, 2021	4/286	7/276	
Spyropoulos, 2021			4/45
Stattin, 2020	0/31		
Voicu, 2021	7/50		

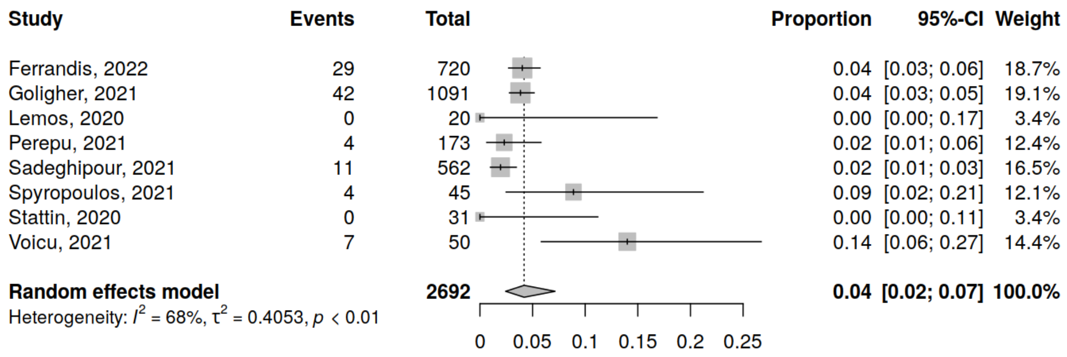

Supplementary Figure S7. Forest plot of major bleeding incidence in COVID-19 patients in the ICU.

Supplementary Table S10. Summary of studies reporting minor bleeding among COVID-19 patients in ICU receiving anticoagulation.

Study (author, year)	Proportion of patients with minor bleeding (n/N)		
	Prophylactic dose	Intermediate dose	Therapeutic dose
Ferrandis, 2022	16/258		21/462
Lemos, 2020	0/10		2/10
Perepu, 2021	6/86	6/87	
Sadeghipour, 2021	5/286	12/276	
Stattin, 2020	0/31		

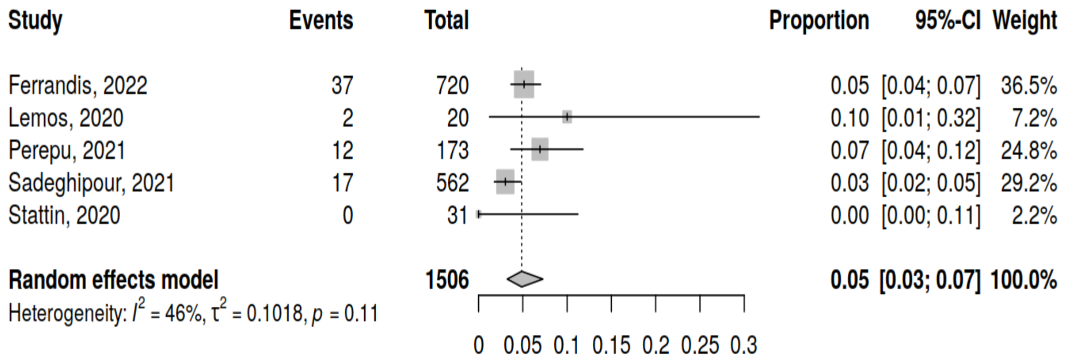

Supplementary Figure S8. Forest plot of minor bleeding incidence in COVID-19 patients in the ICU.

A

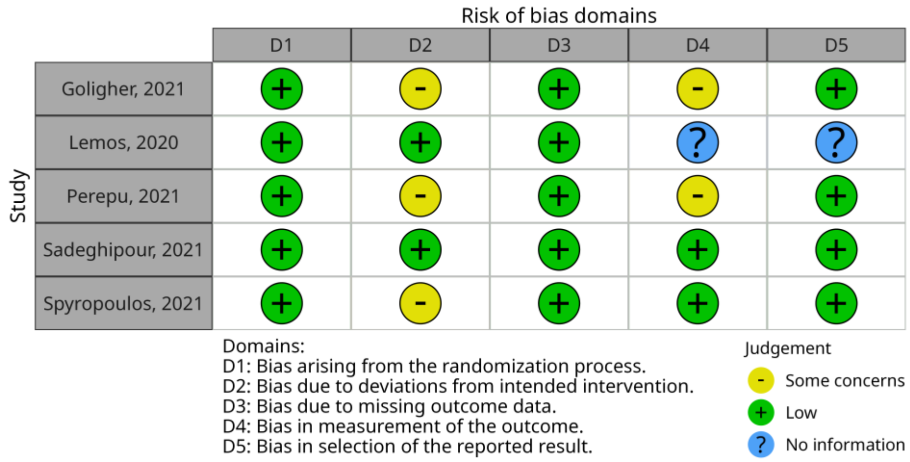

B

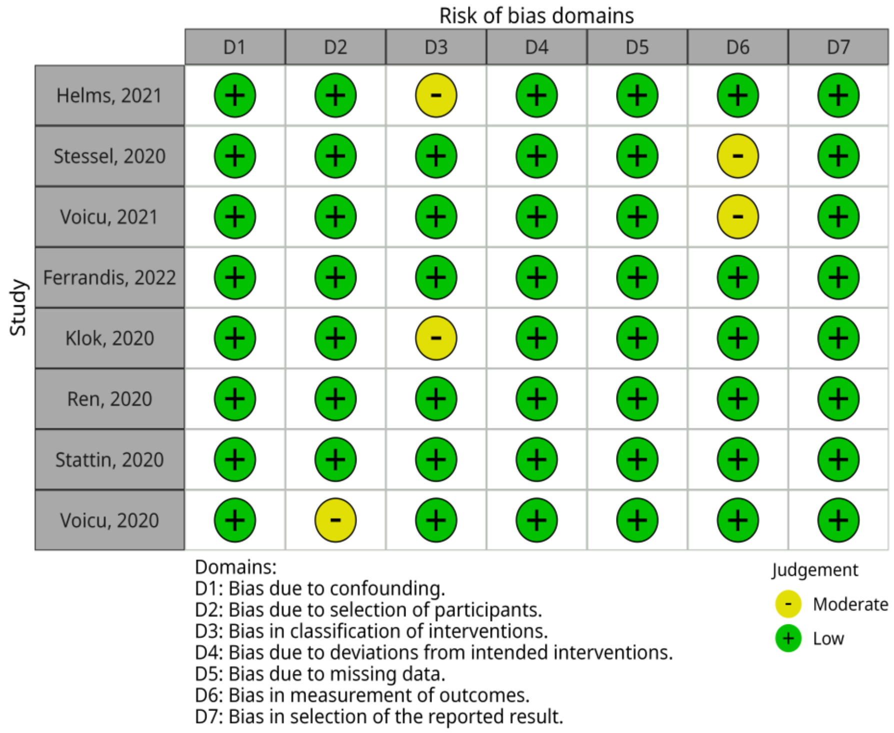

Supplementary Figure S9. Risk-of-bias plots for RCTs (A) and observational studies (B).

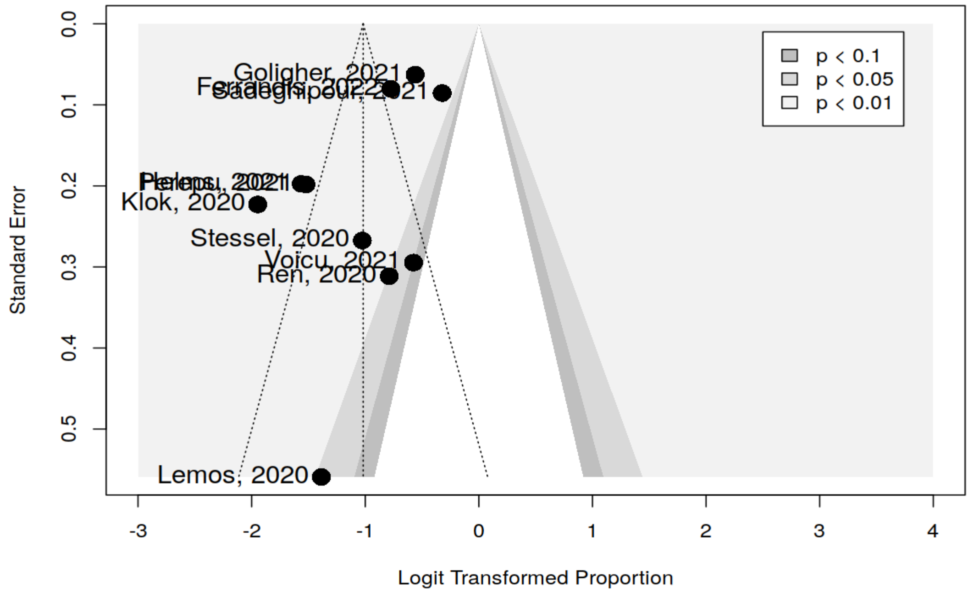

Eggers test:

Intercept	95% CI	t	p-value
-3.38	[-6.37; -0.39]	-2.22	0.057

Supplementary Figure S10. Funnel plot and Egger's test results for the studies of short-term mortality in COVID-19 patients in the ICU.

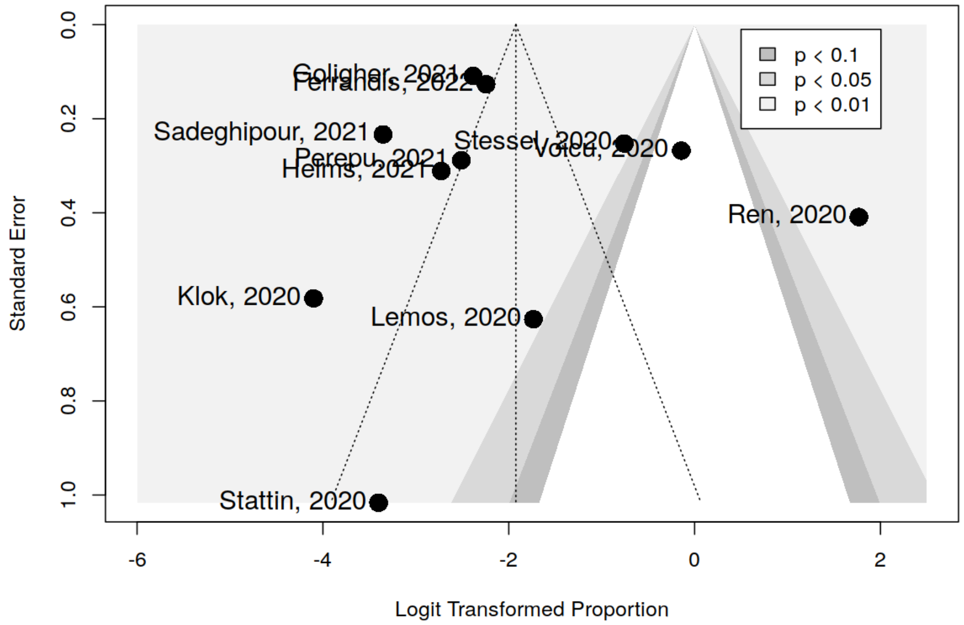

Eggers test:

Intercept	95% CI	t	p-value
1.85	[-3.59; 7.28]	0.67	0.522

Supplementary Figure S11. Funnel plot and Egger's test results for the studies of DVT incidence in COVID-19 patients in the ICU.

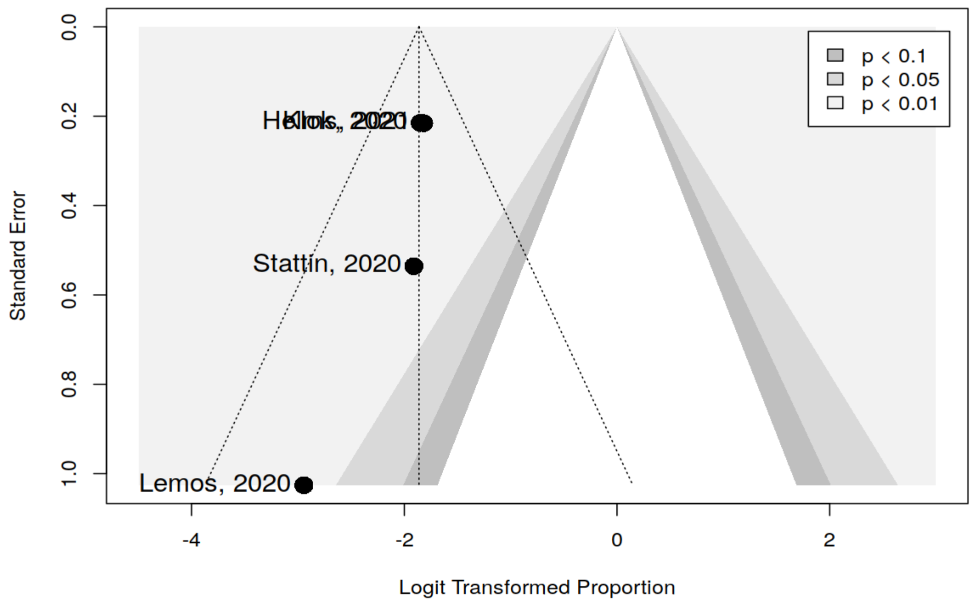

Eggers test:

Intercept	95% CI	t	p-value
-1.09	[-4.19; 2.01]	-0.69	0.541

Supplementary Figure S12. Funnel plot and Egger's test results for the studies of PE incidence in COVID-19 patients in the ICU.

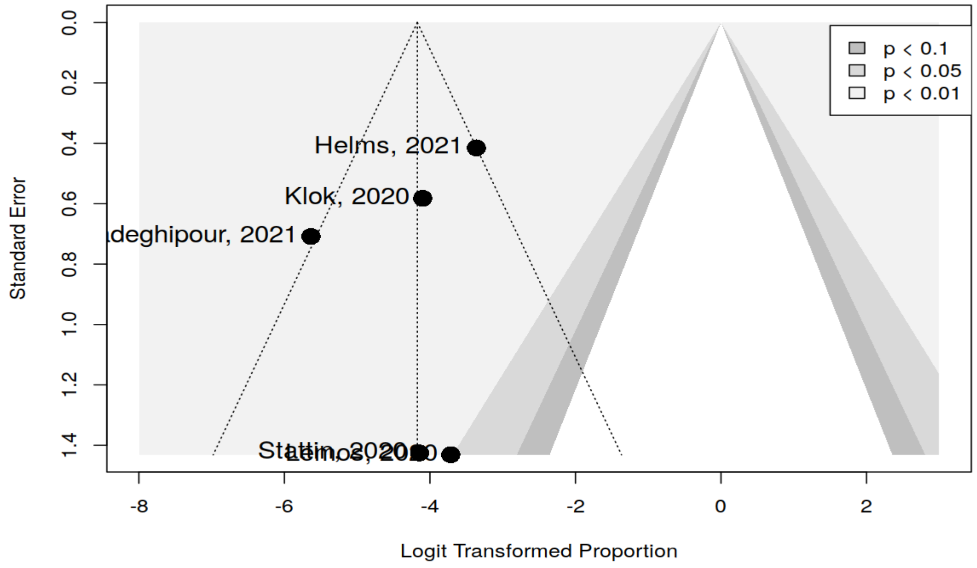

Eggers test:

Intercept	95% CI	t	p-value
-0.98	[-1.77; -0.18]	-2.41	0.137

Supplementary Figure S13. Funnel plot and Egger's test results for the studies of AT incidence in COVID-19 patients in the ICU.

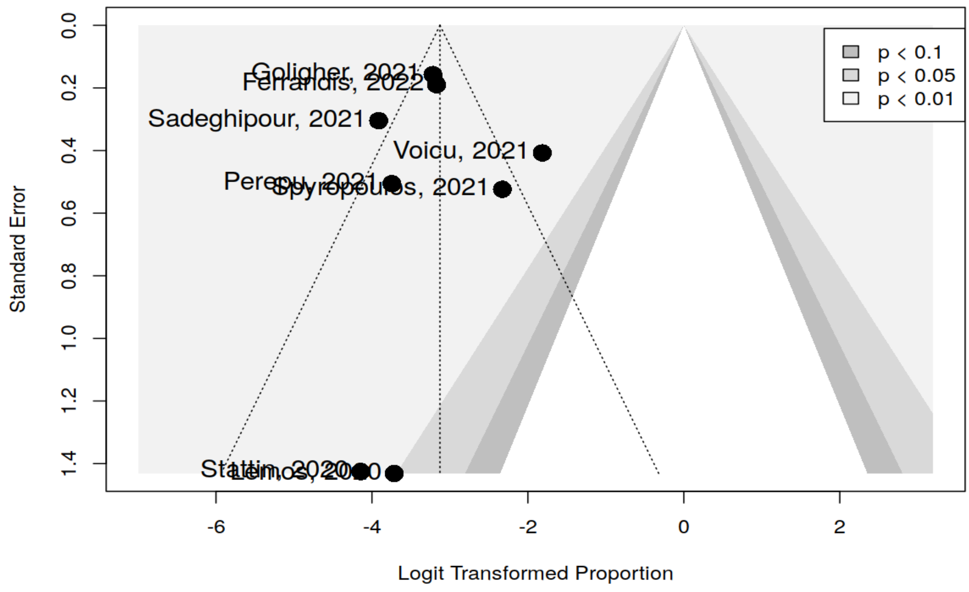

Eggers test:

Intercept	95% CI	t	p-value
0.16	[-2.18; 2.5]	0.14	0.897

Supplementary Figure S14. Funnel plot and Egger's test results for the studies of major bleeding in COVID-19 patients in the ICU.

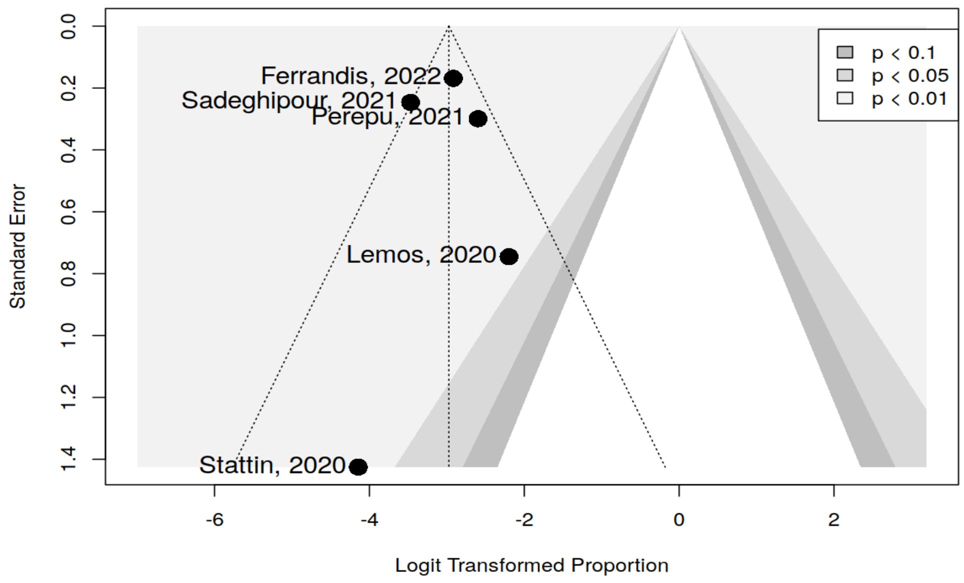

Eggers test:

Intercept	95% CI	t	p-value
0.05	[-2.59; 2.7]	0.04	0.971

Supplementary Figure S15. Funnel plot and Egger's test results for the studies of minor bleeding in COVID-19 patients in the ICU.

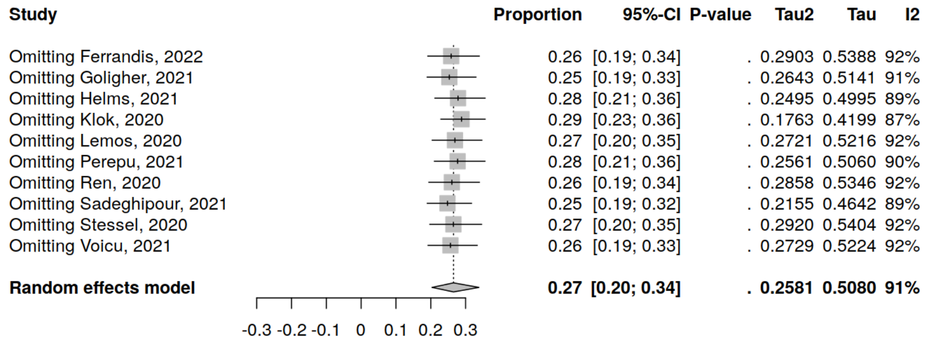

Supplementary Figure S16. Sensitivity analysis of anticoagulation doses influence on short-term mortality in COVID-19 patients in the ICU by the means of the leave-one-out method.

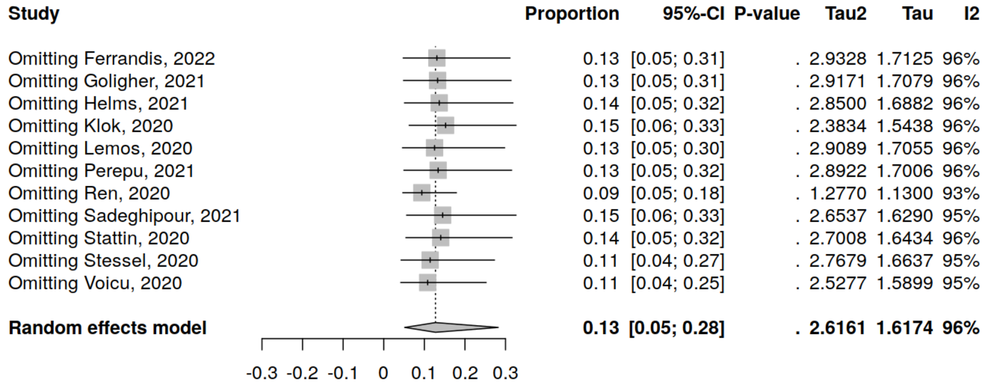

Supplementary Figure S17. Sensitivity analysis of anticoagulation doses influence on DVT incidence in COVID-19 patients in the ICU by the means of the leave-one-out method.

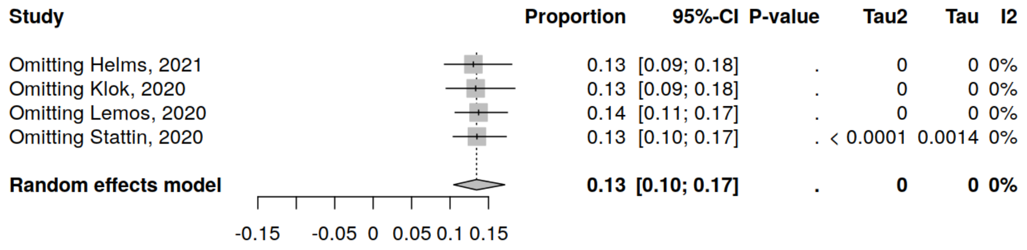

Supplementary Figure S18. Sensitivity analysis of anticoagulation doses influence on PE incidence in COVID-19 patients in the ICU by the means of the leave-one-out method.

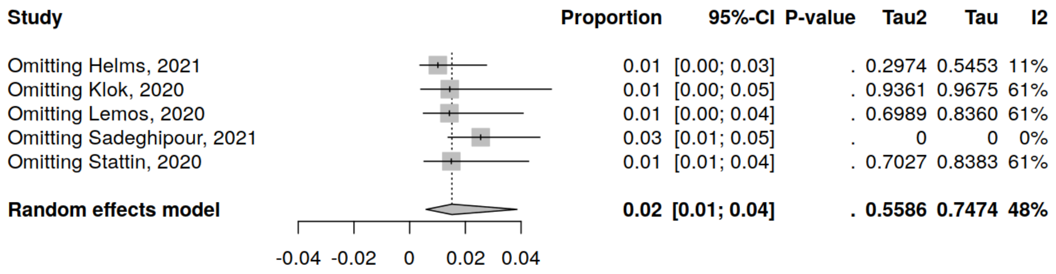

Supplementary Figure S19. Sensitivity analysis of anticoagulation doses influence on AT incidence in COVID-19 patients in the ICU by the means of the leave-one-out method.

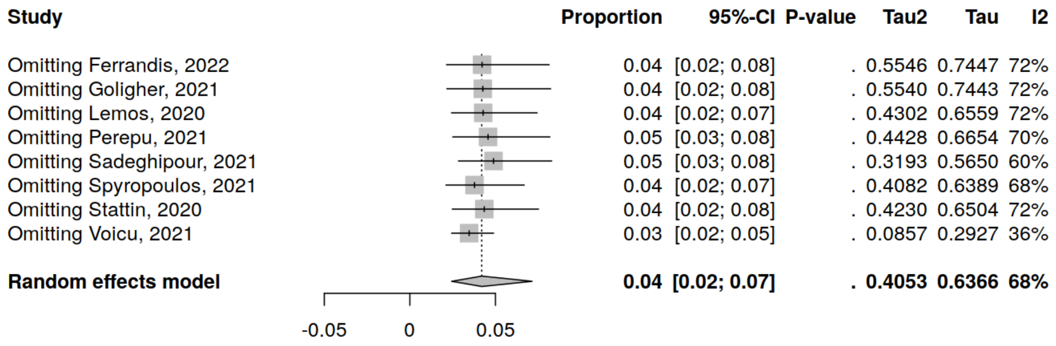

Supplementary Figure S20. Sensitivity analysis of anticoagulation doses influence on major bleeding incidence in COVID-19 patients in the ICU by the means of the leave-one-out method.

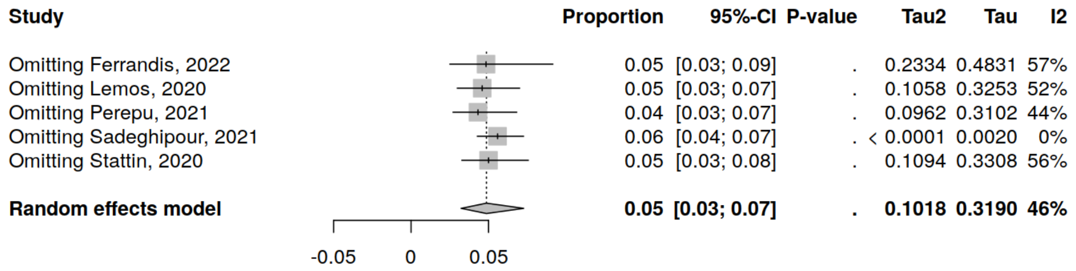

Supplementary Figure S21. Sensitivity analysis of anticoagulation doses influence on minor bleeding incidence in COVID-19 patients in the ICU by the means of the leave-one-out method**.**
